# High‐Voltage Electrolyte Chemistry for Lithium Batteries

**DOI:** 10.1002/smsc.202100107

**Published:** 2022-02-18

**Authors:** Kanglong Guo, Shihan Qi, Huaping Wang, Junda Huang, Mingguang Wu, Yulu Yang, Xiu Li, Yurong Ren, Jianmin Ma

**Affiliations:** ^1^ School of Materials and Energy University of Electronic Science and Technology of China Chengdu Sichuan 611731 China; ^2^ School of Physics and Electronics Hunan University Changsha 410082 Hunan China; ^3^ School of Materials Science and Engineering Jiangsu Collaborative Innovation Center of Photovoltaic Science and Engineering Changzhou University Changzhou 213164 Jiangsu China

**Keywords:** electrolytes, failure mechanism of lithium batteries, high-voltage electrolytes, lithium batteries, electrolyte modification strategies

## Abstract

Lithium batteries are currently the most popular and promising energy storage system, but the current lithium battery technology can no longer meet people's demand for high energy density devices. Increasing the charge cutoff voltage of a lithium battery can greatly increase its energy density. However, as the voltage increases, a series of unfavorable factors emerges in the system, causing the rapid failure of lithium batteries. To overcome these problems and extend the life of high‐voltage lithium batteries, electrolyte modification strategies have been widely adopted. Under this content, this review first introduces the degradation mechanism of lithium batteries under high cutoff voltage, and then presents an overview of the recent progress in the modification of high‐voltage lithium batteries using electrolyte modification strategies. Finally, the future direction of high‐voltage lithium battery electrolytes is also proposed.

## Graphical abstract

High‐voltage lithium batteries have some challenges, e.g., electrolyte decomposition, parasitic oxidation reaction, transition metal dissolution and surface cracks and phase changes in regards with cathodes. In this review, we will overview the recent progress in the modification of high‐voltage lithium batteries using electrolyte modification strategies, and propose future research directions.

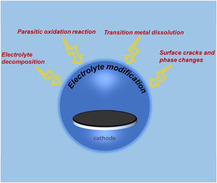

## 1 Introduction

At present, as the concept of carbon neutrality takes root in the hearts of the people and the increasingly serious greenhouse effect, air pollution caused by energy supply urgently needs to be minimized. Compared with the method of burning fossil fuels to obtain energy, the position of rechargeable lithium battery power supply technology with almost no pollution emissions is gradually improving in the field of energy technology.^[^ ^1^ ^]^ The development history of rechargeable lithium‐ion batteries has been since decades. As early as 1991, Sony Corporation developed the first commercial rechargeable lithium‐ion battery. In the following decades, a lot of research aimed at improving the performance of lithium‐ion batteries has made lithium battery technology increasingly mature. Although rechargeable lithium‐ion battery technology has been widely used in our lives, with the increase in the power of portable electronic devices, the desire for long‐range electric vehicles (EVs), and the desire for electrical energy storage for the grids (EESs), the current lithium‐ion battery technology can no longer meet the demand.^[^ ^2^, ^3^, ^4^ ^]^ The state‐of‐the‐art commercial rechargeable lithium‐ion batteries are mainly composed of cathode and anode materials, electrolyte, current collector, and separator. Among these components, the main contribution to the specific capacity is the cathode and anode materials. In terms of anode materials, to pursue a higher specific capacity, researchers have shifted their attention from the traditional carbon‐based anodes to lithium metal.^[^ ^5^ ^]^ This is because lithium metal has the lowest redox potential (−3.04 V, vs standard hydrogen electrode) and low density (0.534 g cm^−3^), resulting in its theoretical specific energy density as high as 3860 mAh g^−1^, which is nearly 10 times that of graphite anode.^[^ ^6^, ^7^, ^8^, ^9^ ^]^ Because lithium metal has extremely high reactivity, using it as an anode will cause additional reactions with general commercial electrolytes, resulting in uneven lithium deposition and dendritic growth, and even safety hazards.^[^ ^10^, ^11^, ^12^ ^]^ Fortunately, many works in recent years have basically solved this problem through a variety of strategies, including surface coatings, porous current collectors, electrolyte modification.^[^ ^13^, ^14^, ^15^, ^16^ ^]^ In contrast to anode materials, the theoretical capacity of cathode materials with the highest specific capacity (such as lithium cobalt oxide, nickel‐rich materials, etc.) is only about 270 mA g^−1^, which greatly prevents the increase in the energy density of the battery. In theory, there are two ways to increase the specific capacity of the cathode. One is to develop a new cathode material with a higher specific capacity (e.g., increasing the nickel content in the layer transition metal oxide cathode material, as the more nickel the cathode can extract more Li^+^ at the same voltage), and the other is to increase the upper‐limit charging voltage of the battery. Obviously, the latter is easier to obtain a high specific capacity. (According to the formula, energy = capacity × voltage) Whereas, an excessively high upper‐limit charging voltage will cause a series of problems in various components of the battery, such as irreversible phase changes, aggravated side reactions, transition metal dissolution, etc., which will make the battery fail prematurely. Not only that, the inert components that were originally thought not to participate in the reaction, such as current collectors, binders, and conductive carbon, will also degrade at a sufficiently high charge cutoff voltage.^[^ ^17^ ^]^ This is an aspect that is easily neglected, but it also leads to rapid battery failure.

**Figure 1:**
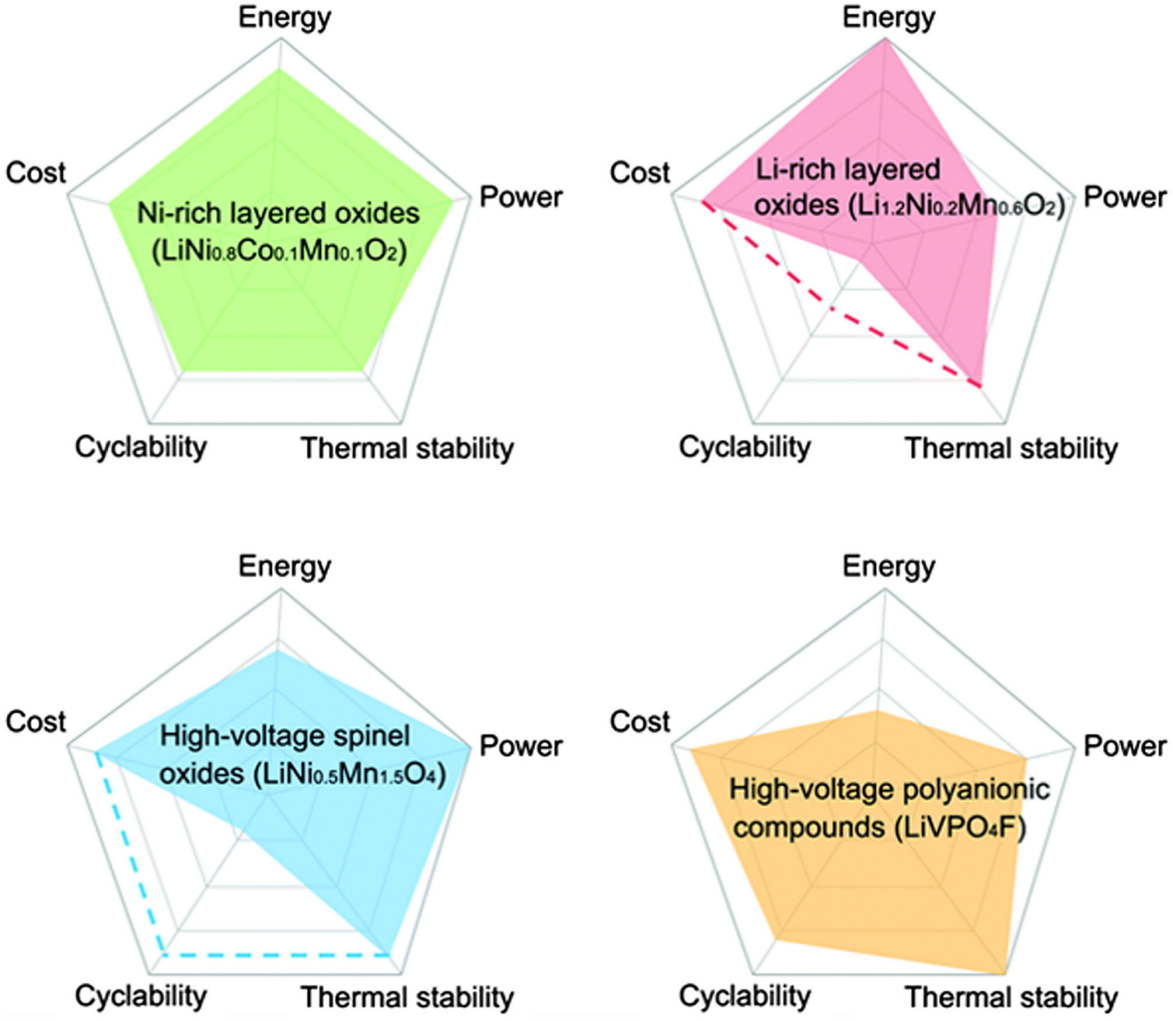
A comparison chart of various high‐voltage cathode materials in terms of energy density, power performance, cycle performance (when commercial carbon‐based cathodes are used), cost, and thermal stability. The dotted line represents the performance obtained in lithium metal half cells. Reproduced with permission.^[^ ^97^ ^]^ Copyright 2017, Royal Society of Chemistry.

Among the many cathode materials, their different characteristics determine their application fields, and some of them have been commercialized. Nickel‐rich cathode material is a kind of cathode material with broad application prospects, which has been adopted by many electric vehicle manufacturers due to its large specific capacity, high working voltage, and excellent high‐power output performance.^[^ ^18^ ^]^ In addition, increasing the proportion of nickel in the material can increase the specific capacity. LiCoO_2_ is the first cathode material developed. Its volumetric energy density is higher than other cathode materials, and its theoretical specific capacity is particularly high. Portable electronic devices need to store as much energy as possible in a limited volume, so LiCoO_2_ is widely used in the batteries of portable electronic devices. LiFePO_4_ is a cathode material that has been developed rapidly and practically. It is mainly used in energy storage equipment, high‐power electric tools, and light electric vehicles. The most competitive advantage is its good cycle stability (over 2000 times of charging and discharging), and good rate performance. The most important thing is that it is safe, nontoxic, and low in cost. At the same time, low capacity and low bulk density hinder its further development. LiMnO_2_ is a cathode material with a spinel structure. Due to its three‐dimensional lithium ions channel, it cannot damage the structure under multiple cycles. Its characteristics are cheap, pollution‐free, and high discharge voltage platform. As people's demand for battery energy density continues to increase, some cathode materials suitable for high voltage have been developed. Among them, candidates for high‐voltage cathode materials worthy of high hope include nickel‐rich layered oxides (LiNi_*x*_Co_*y*_Mn_*z*_O_2_ and LiNi_*x*_Co_*y*_Al_*z*_O_2_ (*x* + *y* + *z* = 1)), lithium‐rich layered oxides (Li_1+*x*_M_1–*x*_O_2_, M = Mn, Ni, Co), high‐voltage spinel oxides (LiNi_0.5_Mn_1.5_O_4_), and high‐voltage polyanionic compounds (phosphates, sulfates, silicates, etc.).^[^ ^19^ ^]^ **Figure** **1** shows the energy density, power, cyclability, cost, and thermal stability of various high‐voltage cathode materials. Under high‐voltage conditions (usually higher than 4.2 V), these cathode materials can generally reach a specific capacity higher than 200 mAh g^−1^, although there may be a risk of electrolyte decomposition or irreversible phase change of the material. In recent years, great effort has been made to solve the problem of instability at high voltage, such as electrolyte modification,^[^ ^20^, ^21^ ^]^ electrode surface coating,^[^ ^22^, ^23^, ^24^ ^]^ and doping of elements in the electrode composition,^[^ ^25^, ^26^ ^]^ etc.

In this review, we first discussed the mechanism of battery degradation induced by increasing the upper charging voltage. Different from other reviews, this review also introduces the use of different electrolyte modification strategies to improve lithium batteries at high cutoff voltage. Then, it briefly reviews the relevant work in recent years, and finally puts forward a summary and prospect.

## 2 Failure Mechanism Under High Voltage

### 2.1 Electrolyte Decomposition

**Figure 2:**
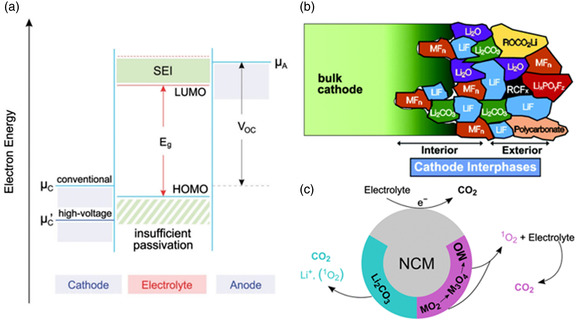
a) Schematic diagram of the relationship between the operating voltage and the electrochemical window of the electrolyte in the open‐circuit state. b) Diagram of electrolyte decomposition products and microstructure on the cathode surface. c). Schematic diagram of the mechanism of CO_2_ release on an NCM cathode material. a) Reproduced with permission.^[^ ^97^ ^]^ Copyright 2017, Royal Society of Chemistry. b) Reproduced under the terms of the CC‐BY 4.0 license.^[^ ^98^ ^]^ Copyright 2017, The Authors, published by Springer Nature. c) Reproduced with permission.^[^ ^33^ ^]^ Copyright 2018, American Chemical Society.

As we all know, when a newly assembled battery is charged for the first time, the electrolyte on the anode and cathode surfaces are reduced and oxidized, respectively, forming a passivation film on the electrode surface. The interface film formed on the anode is called solid electrolyte interphase (SEI), and the interface film formed on the cathode is called cathode electrolyte interphase (CEI). In general, the passivation layer generated on the electrode surface is expected to satisfy three characteristics. First, the impedance is small, that is, it can conduct Li^+^ quickly. Second, its high resistivity can effectively insulate the contact between the electrode and the electrolyte, and reduce the maximum thickness of the electron tunneling effect, thus preventing further thickening of the interface film. Third, its mechanical strength should be large, and it can withstand the volume changes of electrode materials in the process of lithium removal and lithium insertion without cracking. Which component of the electrolyte takes precedence in redox reactions can be determined by testing its redox potential, which is essentially determined by the energy orbital of the molecule. The higher the highest occupied molecular orbital (HOMO) of a molecule, the lower its oxidation potential, the easier it is to lose electrons and be oxidized. If the lowest unoccupied molecular orbital (LUMO) of a molecule is lower, it has a higher reduction potential, and it is easier for it to obtain electrons and be reduced.^[^ ^27^ ^]^ HOMO and LUMO of each molecule can be calculated. By comparing their levels, we can roughly judge which component reacts first. The potential region consisting of the reduction potential and oxidation potential of the electrolyte is called the redox window of the electrolyte. Ideally, the redox window of the electrode solution should be lower than the reduction potential of the anode and higher than the oxidation potential of the cathode, meaning that the electrolyte will not decompose and will only serve to transfer charge. However, in reality, the decomposition of the electrolyte is inevitable, and there is always a small part of the electrolyte components on both sides of the cathode and anode that tend to be oxidized and reduced. The electrolyte component forming the interphase is mainly solvent, and some Li^+^ will be consumed during this process, which will consume the limited Li source in the battery and greatly reduce the reversible capacity. **Figure** **2**a illustrates the relationship between the energy levels of the electrons in the electrode and the operating voltage, as well as the relationship between the electrochemical window of the electrode solution and the formation of the passivation film. Since the electrons in the lithium anode or graphite anode are easily given out, and the reduction potential of almost all electrolytes are above their redox potential, it is inevitable to form SEI on the surface of the anode by consuming the electrolyte. Film formation on the cathode is more difficult to inhibit the decomposition of the electrolyte than on the anode, because more physical and chemical changes occur on the cathode surface than on the anode, which is especially evident when the high‐voltage cathode is used and charged to a high‐voltage state. This is also the reason for the low coulomb efficiency of the full cell in the cycle. Currently, commercial electrolytes are mainly carbonate‐based electrolytes, which are considered to be unable to circulate for a long time at a voltage greater than 4.3 V.^[^ ^28^ ^]^ Electrolyte decomposition products are diverse and stack haphazardly on the surface of the cathode. As shown in Figure 2b, this is a complex evolution in battery operation, many different kinds of products are jumbled together to form what is known as a Mosaic model, and their structure and content affect the battery's performance. In fact, the cathode cannot be considered as an inert component in this process, which significantly affects the decomposition of the electrolyte, this is the source of the parasitic reactions mentioned later. Taking LCO under the condition of high voltage as an example, under the charging voltage greater than 4.2 V, more than 50% Li^+^ will be released from LCO and a large amount of Co^3+^ will be oxidized to Co^4+^. In an unstable oxidation state, Co^4+^ is easy to dissolve from LCO into the electrolyte, which will destroy the structure of LCO and lead to rapid capacity attenuation. To make matters worse, the high oxidation state of Co^4+^ catalyzes the decomposition of the electrolyte, eliminating too much electrolyte and causing premature battery failure and even safety problems.

### 2.2 Parasitic Oxidation Reaction

**Figure 3:**
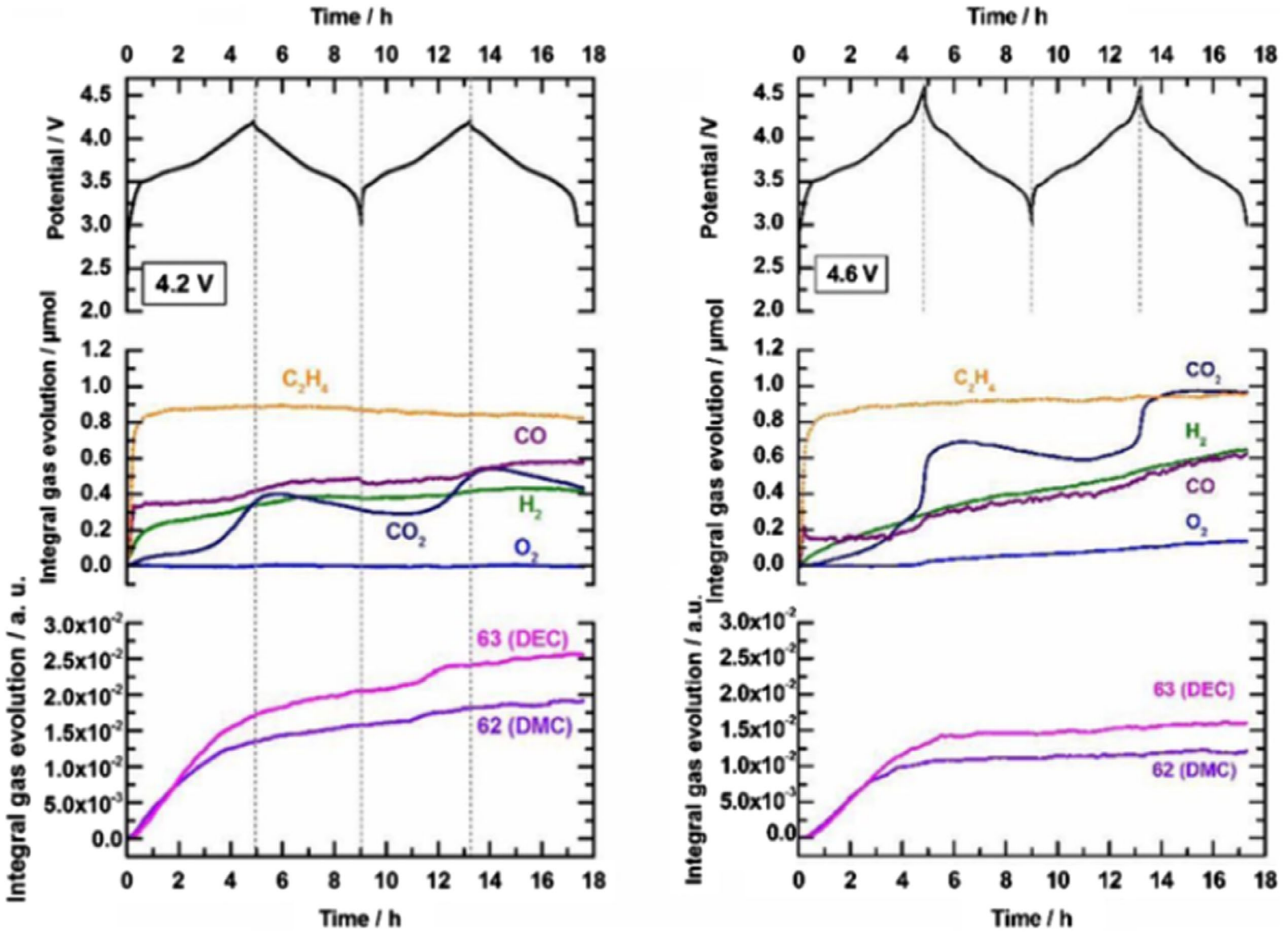
The online electrochemical massspectroscopy (OEMS) analysis of Gr || NCM811 batteries in the first two cycles with charging cutoff voltages of 4.2 and 4.6 V, respectively, the electrolyte used was 1 m LiPF_6_ in EC:EMC = 3:7 (by volume). Reproduced under the terms of the CC‐BY 4.0 license.^[^ ^32^ ^]^ Copyright 2019, The Authors, published by ECS.

**Figure 4:**
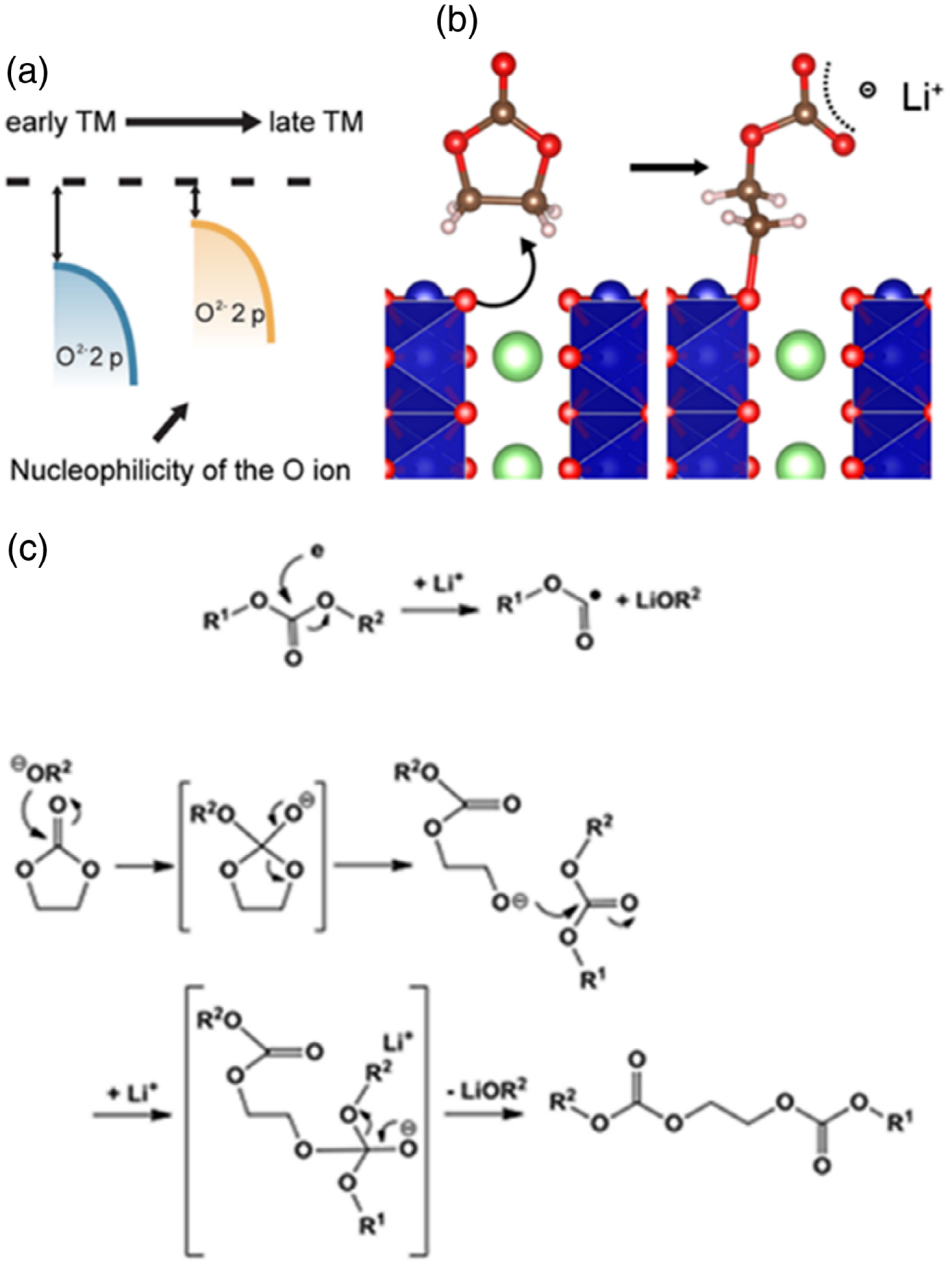
Illustration of nucleophilic attack on aprotic solvent by oxygen ions on metal oxide surfaces. a) The oxygen ions on transition metal oxides with larger atomic numbers are more nucleophilic. b) Schematic diagram of nucleophilic attack of EC molecule by oxygen ions on the surface of lithium layered oxide. c) The possible pathway of trans‐esterification between EC and DEC, here *R* ^1^ = *R* ^2^ = Ethyl. a) Reproduced with permission.^[^ ^36^ ^]^ Copyright 2015, American Chemical Society. b) Reproduced with permission.^[^ ^37^ ^]^ Copyright 2018, Elsevier B.V.

Parasitic oxidation reaction is another important factor affecting battery performance degradation at high voltage. Because passivation of the cathode is more difficult than passivation of the anode, the electrolyte on the highly delithiated cathode is prone to continuous oxidation, although this process is slow. The reason is thought to be that the passivation film on the surface of the cathode does not effectively cover the cathode, resulting in the electrolyte molecules being taken away by the cathode material, so this process is called parasitic oxidation.^[^ ^29^ ^]^ The continuous parasitic oxidation reaction under high voltage will cause many harms that lead to the premature failure of lithium batteries. When the lithium source is limited, the parasitic reaction will continue to consume the active lithium ions in the cathode material, causing a sharp decline in the reversible capacity. And the organic or inorganic components produced by these reactions will accumulate unevenly on the surface of the cathode, increasing the impedance. In addition, when using carbonate solvents, parasitic reactions are often accompanied by gases, the products of which are CO_2_, CO, O_2_, CH_4_, and H_2_. These gases will block the electrode surface, hinder the migration of lithium ions, and cause uneven current distribution. The oxygen in the gas may come from the electrolyte oxidized and decomposed by oxygen substances escaping from transition metal oxides in the form of non‐oxygen molecules (singlet oxygen or reactive oxygen, ^1^O_2_), which also causes structural instability.^[^ ^30^ ^]^ What's more serious is that the generated gas will increase the pressure in the battery and cause the volume of the battery to expand, this phenomenon is particularly obvious in the soft package battery. It is certain that gas will be generated on both the cathode and anode. The gas produced at low voltage mainly comes from the formation and reaction of SEI on the anode, while the evolution of gas at high voltage is mainly related to the oxidation of electrolyte on the cathode,^[^ ^31^ ^]^ and in the high‐voltage system, the gas mainly comes from the decomposition of electrolyte on the positive electrode by various mechanisms. N. Laszczynski et al. studied the gas evolution of a high‐voltage NCM811 || graphite negative battery using 1 m LiPF_6_ dissolved in EC and EMC (3:7 volume ratio) electrolyte. As a result, batteries cycled at 4.6 V produce significantly more gas than those produced at 4.2 V, as shown in **Figure** **3**.^[^ ^32^ ^]^ This can be attributed to the fact that at higher voltages, the degree of parasitic reaction on the cathode surface is more intense, resulting in more gas products. Hatsukade T and his colleagues studied the origin of CO_2_ generated in parasitic reactions using isotope labeling and electrolyte substitution in differential electro‐chemical mass spectrometry–differential electrochemical infrared spectroscopy measurements.^[^ ^33^ ^]^ Their results can be briefly illustrated in Figure 2c. The production of gas products is related to many factors. First, under high potential, the electrochemical decomposition of Li_2_CO_3_ on the surface of the NCM helps to release CO_2_,^[^ ^34^ ^]^ especially during the first charge. Second, CO_2_ produced by electrolyte decomposition is dominant. This is inseparable from the fact that NCM materials suffer from H2 to H3 phase transition when at high states of charge (SOC) about 80%, resulting in the release of lattice oxygen. The released reactive oxygen species (^1^O_2_) will continuously chemically oxidize the carbonate solvents in a number of possible ways to produce harmful substances such as CO_2_ and protons.^[^ ^35^ ^]^ When the cathode material is in a high state of charge, it is in an unstable and highly delithiated state. The oxygen ion on its surface can easily donate two electrons to form a chemical bond with the carbonate solvent molecule, thereby catalyzing the decomposition of the electrolyte. The process is called nucleophilic attack. And the nucleophilic attack ability of oxygen ions on the surface of metal oxides will increase with the increase of electronegativity (e.g., Ni > Co > Mn), and the greater covalency of M–O bonds. This trend is described in **Figure** **4**a, as the number of transition metal atoms increases, the 2p energy level of oxygen moves closer to the Fermi level, causing its energy level to be unstable and thus showing increased nucleophilicity. As shown in Figure 4b, the EC molecule undergoes a ring‐opening reaction after the nucleophilic attack, which will generate products including Li_2_CO_3_, hemicarbonate, polycarbonate, alkoxide, etc. In the end, this will undoubtedly increase the organic components in the CEI, causing the thickening of the CEI and the increase in impedance.^[^ ^36^ ^]^ In addition to the reaction between solvent and electrode, parasitic reactions also occur between different solvent molecules. The products produced by the reaction are often dissolved in the bulk electrolyte rather than deposited on the electrode surface, which leads to this kind of reaction has not received too much attention. For example, in the trans esterification reaction between EC and DEC, lithium alkoxide obtained from carbonate will nucleophilic attack another carbonate molecule (Figure 4c), and the generated diethyl 2,5‐dioxahexane dicarboxylate (DEDOHC) has been proved to affect the impedance of lithium battery.^[^ ^37^ ^]^ In recent work, Rinkel et al. compared the composition of the electrolyte after electrolysis and cycling in battery, respectively. They found that these components were completely different, which proved that the oxidation on the cathode surface is more chemical oxidation (mainly due to the singlet oxygen released from the cathode material) than electrochemical oxidation. Lithium‐ion conductive glass‐ceramic is used to separate the anode and cathode compartments, so as to avoid the “cross‐talk” of electrolyte decomposition products from one side to the other and interfere with the oxidation products produced by analysis.^[^ ^38^ ^]^

### 2.3 Transition Metal Dissolution

**Figure 5:**
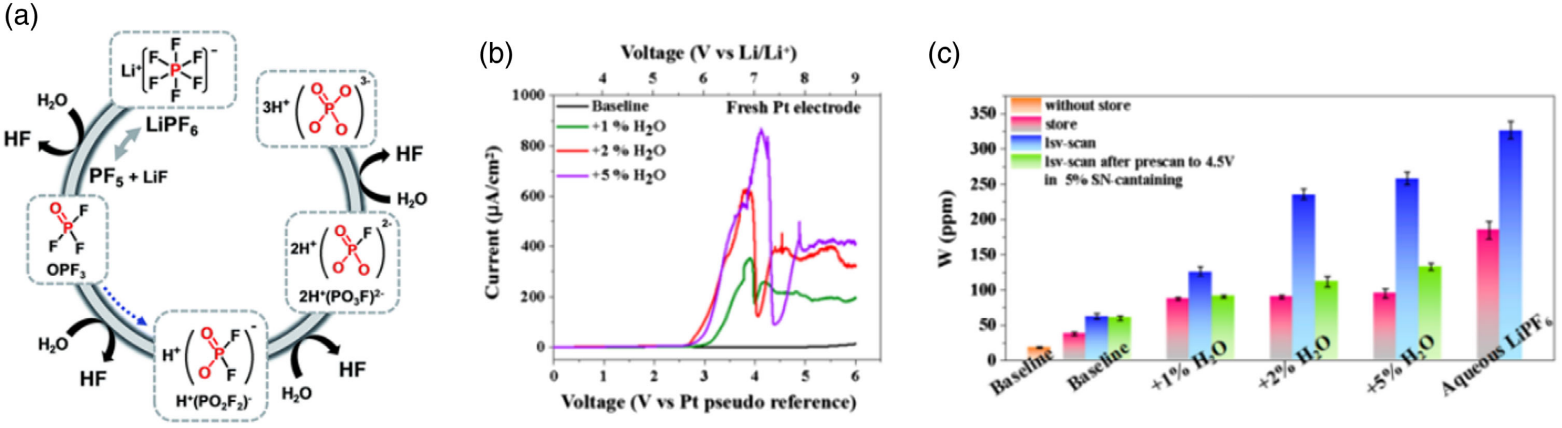
a) In the electrolyte with LiPF_6_ as the lithium salt, a series of hydrolysis reactions occur in the presence of trace amounts of water. b) Linear voltammetry curves of LiPF_6_ salt electrolyte with different water content. c) The content of HF produced after the electrolyte is stored at room temperature or after linear scanning. a) Reproduced with permission.^[^ ^39^ ^]^ Copyright 2015, Royal Society of Chemistry. b,c) Reproduced with permission.^[^ ^41^ ^]^ Copyright 2021, American Chemical Society.

**Figure 6:**
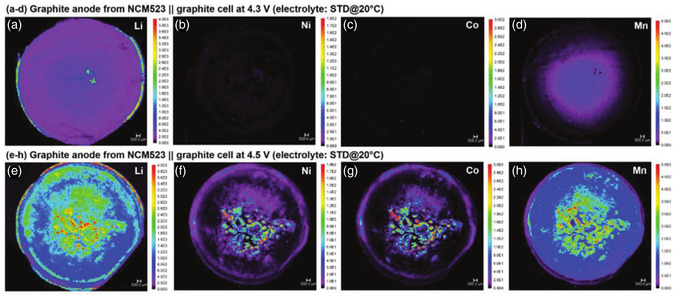
LA‐ICP‐MS analysis of graphite anode of NCM523 || graphite full battery after 100 charge–discharge cycles. a–d) The battery runs at 4.3 V; e–h) The battery runs at 4.5 V. Reproduced with permission.^[^ ^42^ ^]^ Copyright 2021, Wiley‐VCH.

The presence of acidic substances such as hydrogen fluoride is an important factor leading to the degradation of battery performance. It can destroy the solid electrolyte interface and release gas, corrode the current collector, catalyze the decomposition of the electrolyte into polycarbonate, and accelerate the dissolution of transition metals. As the most commonly used lithium salt, LiPF_6_ has the defects of poor thermal and chemical stability. HF and PF_5_ (a strong Lewis acid) have been confirmed to come from its hydrolysis and thermal decomposition, respectively. As shown in **Figure** **5**a, the main reactions involved in this process are LiPF_6_ → LiF + PF_5_ and PF_5_ + H_2_O → POF_3_ + 2HF.^[^ ^39^ ^]^ In the high potential state, ${{\text{PF}}_{6}}^{-}$anion can also extract hydrogen atoms from carbonate molecules and oxidize them (${{\text{PF}}_{6}}^{-}$+ R‐H → PF_5_ + HF + $R^{•}$).^[^ ^40^ ^]^ Water may be produced at high voltages by continuous oxidation of the carbonate solvent with singlet oxygen released from the cathode material,^[^ ^38^ ^]^ even if the water content in the possible electrolyte is negligible. Moreover, LiPF_6_ hydrolysis accelerates with the increase of voltage. Liu et al. found that in the electrolyte with a lot of water, the oxidation peak current of the electrolyte will become larger, indicating that the presence of water does accelerate the decomposition of the electrolyte. At the same time, they also found that the HF generated under high voltage is significantly higher than that of storage at room temperature (Figure 5b,c). In addition, they revealed that HF is derived from LiPF_6_ more than PF_5_, as previously thought.^[^ ^41^ ^]^ When the transition metal is dissolved in the electrolyte, like the shuttle effect in the lithium–sulfur battery, it will be reduced on the lithium anode, causing uneven lithium deposition on the lithium anode and severe dendrite growth, which will cause the battery to quickly fail, and even cause safety hazards. Sven Klein et al. observed that the dissolution of transition metals in NCM523 || graphite batteries was severely increased at higher voltages by laser ablation‐inductively coupled plasma‐mass spectrometry (LA‐ICP‐MS) analyses, especially the dissolution of Mn cations was more serious than that of Ni and Co cations (**Figure** **6**).^[^ ^42^ ^]^ It is worth noting that the attachment position of these transition metals on the graphite anode and the growth position of the lithium dendrites are highly coincident, which proves that the leaching of transition metals can worsen the lithium deposition of the lithium anode or the graphite anode. Transition metals will dissolve intensified as the voltage rises. In addition to causing the growth of lithium dendrites on the anode, the micropores on the separator may also be blocked. This undoubtedly hinders the transmission of Li^+^ on the separator, leading to an increase in battery impedance. In addition to the dissolution of transition metals caused by HF corrosion of the cathode material, the manganese disproportionation reaction may also be a way to cause Mn cations to dissolve into the electrolyte, according to the formula: 2Mn^3+^ → Mn^2+^ + Mn^4+,^ because Mn cations are found at a lower electrode voltage at the cathode.^[^ ^43^ ^]^

### 2.4 Surface Cracks and Phase Changes

**Figure 7:**
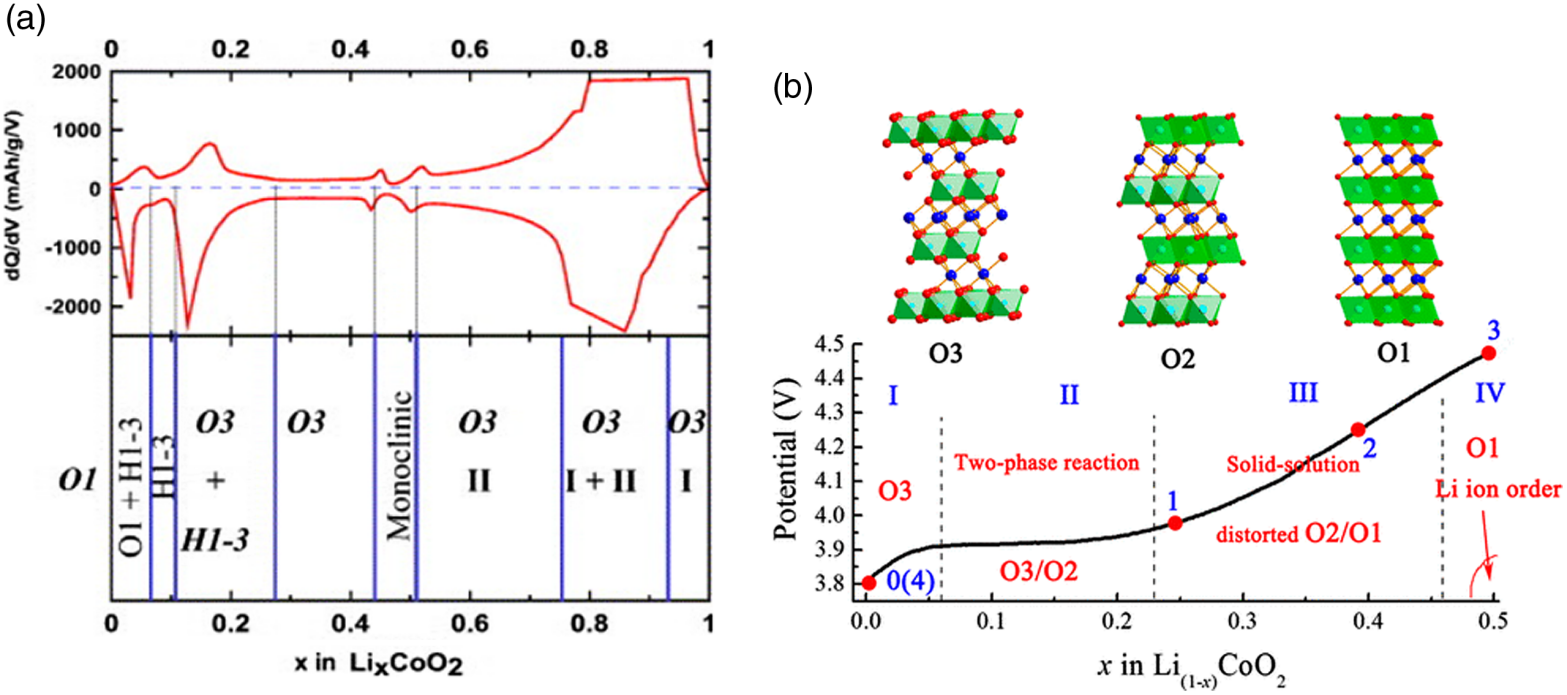
a) The relationship between the differential capacity in Li_*x*_CoO_2_ and the Li concentration and the phase diagram of Li_*x*_CoO_2_; b) Phase diagram of Li_(1–*x*)_CoO_2_ (0 ≤ *x* ≤ 0.50) nanoparticles. The number symbols 0–4 represent the sampling points of scanning transmission electron microscopy observation: 0) original LiCoO_2_, 1) charge to about 3.9 V, 2) charge to 4.2 V, 3) charge to 4.5 V, 4) restore to 3.0 V. a) Reproduced with permission.^[^ ^99^ ^]^ Copyright 2003, Elsevier Science Ltd. b) Reproduced with permission.^[^ ^100^ ^]^ Copyright 2012, American Chemical Society.

As the charge cutoff voltage increases, more active lithium is deintercalated, so the highly delithiated structure becomes more unstable. Taking the successfully commercialized LCO as an example. Ohzuku et al. first discovered the irreversible phase transition of Li_*x*_CoO_2_ (0 < *x* < 0.25) at a voltage exceeding 4.5 V.^[^ ^44^ ^]^ As more lithium is released, the material undergoes a secondary phase transition, the C axis shrinks, and the stress will cause the cathode particles to break, lose electronic contact with the current collector, increase battery polarization, and reduce capacity. As shown in **Figure** **7**, a series of phase transitions occur at the positive electrode of the LCO during charging. First, the H1‐3 phase is shown to be a mixed main structure between rhomboid LCO and hexagonal CoO_2_; second, when the lithium content is 0.19 < *x* < 0.33, O3 and H1‐3 phase coexist, when × is less than 0.12, H1‐3 coexists with the O1 phase; finally, when × in LixCoO_2_ is between 0.12 and 0.19, the phase can remain stable. The transition from the O3 phase to the H1‐3 phase occurs at 4.55 V, and the transition from the H1‐3 phase to the O1 phase occurs at 4.63 V.^[^ ^45^ ^]^ This indicates that the phase transition of the layered oxide cathode material during charge and discharge is an inherent and inevitable property, and causes serious damage to the reversible capacity of the battery.

**Figure 8:**
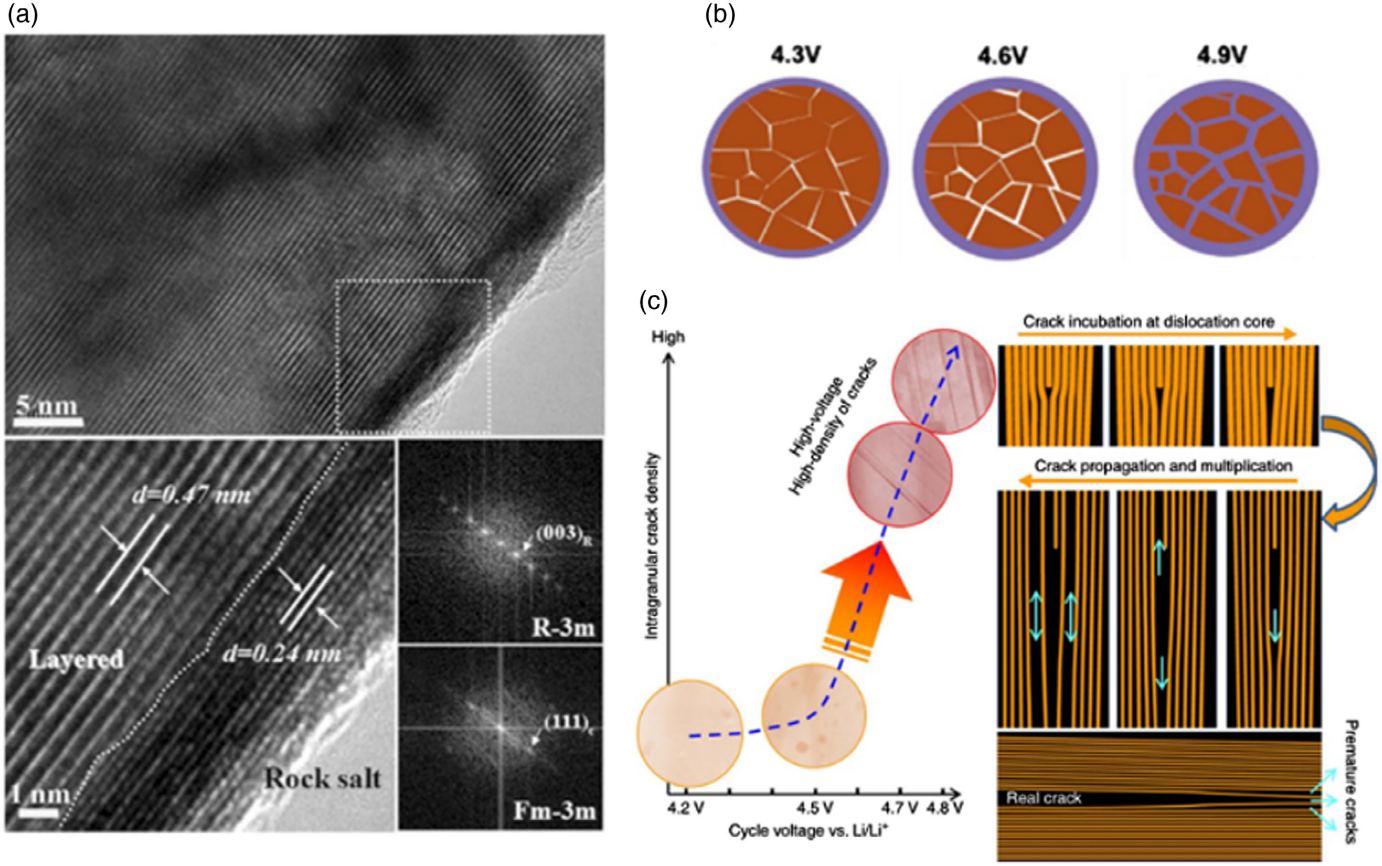
a) Phase transition of NCM811 cathode near surface and bulk. b) The change of microcracks when NCM523 is initially overcharged to 4.9 V. c) Schematic diagram of dislocation assisted crack generation. a) Reproduced with permission.^[^ ^47^ ^]^ Copyright 2016, IOP Publishing. b) Reproduced with permission.^[^ ^101^ ^]^ Copyright 2019, Wiley‐VCH. c) Reproduced under the terms of the CC‐BY 4.0 license.^[^ ^48^ ^]^ Copyright 2017, The Authors, published by Springer Nature.

After the electrolyte interacts with the cathode material, the cathode surface will undergo surface reconstruction, resulting in a layered‐spinel‐rock salt layer structure, which seems to be a compromise of the cathode material to adapt to this environment. The layered‐spinel‐rock salt phase transition is the inevitable result of the migration of transition metal ions, the shear of the atomic layer, and the oxygen evolution reaction. It usually occurs in highly delithiated cathode materials. This crystal rearrangement phenomenon seems to be the inherent properties inside the structure, which are also closely related to the evolution of the electrode/electrolyte interface.^[^ ^46^ ^]^ When the phase transition occurs, the inner body of the cathode particles cannot be quickly consumed due to oxygen, and the oxygen evolution reaction is kinetically hindered. The reactive oxygen generated on the surface is quickly consumed by the electrolyte solvent, which promotes the oxygen evolution reaction. Therefore, the layered‐spinel‐rock salt phase transition near the crack surface is more serious than the other parts of the cathode particles (**Figure** **8**a).^[^ ^47^ ^]^ Since the decomposition products of the rock salt phase and the solvent neither conduct electricity nor have high ionic conductivity, their accumulation results in the formation of a thick and high‐impedance interface layer on the surface.

Repeated lithium removal/lithium insertion processes will cause different crystal deformations on different crystal axes, and macroscopically, the particles will be subjected to anisotropic mechanical stress, which will eventually lead to cracks in the cathode particles. When the cutoff charging voltage is increased, more lithium is extracted, and the cracks on the cathode particles will be more obvious (Figure 8b). To make matters worse, when cracks occur on the surface of the cathode, the electrolyte will penetrate into the particles and react with the active materials inside, resulting in a high‐impedance, uneven surface film. This will cause the uneven diffusion of Li^+^ on the surface of the cathode, resulting in a greater stress difference in all directions, and further increasing the degree of cracks, which is equivalent to a vicious circle. Some scholars also believe that this cracking starts from the internal dislocation of the crystal, rather than from the outside to the inside, as shown in Figure 8c.^[^ ^48^ ^]^ Therefore, they believe that maintaining the structural stability of the material is the key to the operation of layered cathode materials.

### 2.5 Other Inert Components in the Battery at High Voltage

**Figure 9:**
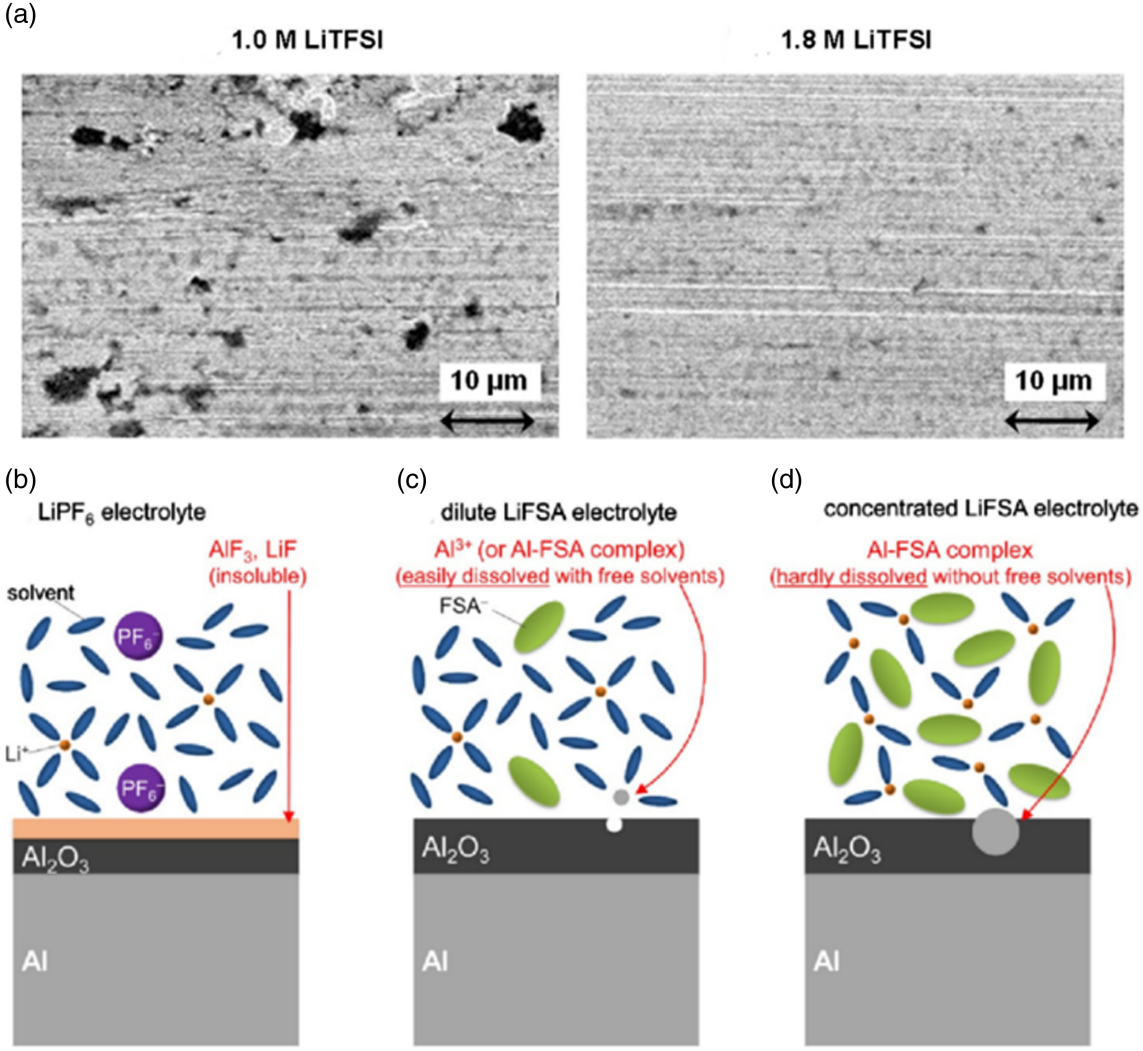
a) SEM image of Al current collector after applying voltage in different concentrations of electrolyte. b–d) Schematic diagram of Al electrode behavior in different electrolytes. a) Reproduced with permission.^[^ ^52^ ^]^ Copyright 2012, Elsevier B.V. b–d) Reproduced with permission.^[^ ^54^ ^]^ Copyright 2015, Wiley‐VCH.

The degradation reactions mentioned earlier mainly occur at the interface between electrolyte and electrode. Beyond that, other inactive materials also react with the electrolyte at high potential. Normally, the cathode material is coated on the Al foil current collector, so the Al foil will also be immersed in the electrolyte and will be corroded by acid present in the electrolyte, resulting in poor contact with the cathode particles and decreased conductivity. Although the original Al_2_O_3_ passivation layer on the Al surface can be eroded to form a passivation layer containing AlF_3_ and LiF in most cases. Especially, under high‐voltage conditions, the passivation layer is still prone to pitting corrosion, leading to degradation of the passivation film, thereby accelerating the dissolution of the Al current collector, and even causing the active material to fall off.^[^ ^17^ ^]^ In addition, the lithium salt used is associated with the corrosion of Al foil. The lithium salt in the electrolyte either lacks fluorine (such as lithium perchlorate, LiClO_4_)^[^ ^49^ ^]^ or exhibits high compatibility with moisture, that is, greater stability (such as lithium bistrifluoromethanesulfonimide, LiTFSI), which will cause oxidation dissolution of Al at high potential. While other lithium salts such as LiPF_6_^[^ ^50^ ^]^ and LiBF_4_^[^ ^51^ ^]^ can significantly inhibit Al foil corrosion by providing F^−^ to form insoluble AlF_3_ and LiF, so in practical use, they are the most widely used lithium salts. The study by Matsumoto et al. showed that when 1M LiTFSI is dissolved in EC/DEC (3:7, by volume ratio) electrolyte, when the voltage rises to 3.7 V, the Al current collector is rapidly oxidized. However, after the lithium salt concentration was increased to 1.8M, the corrosion of the Al current collector was significantly improved (**Figure** **9**a,b).^[^ ^52^ ^]^ Wang et al. also observed the same result after increasing the concentration of LiFSI.^[^ ^53^ ^]^ This may be attributed to the effective protection of the Al collector by anion‐derived passivation films. Yamada et al. explained in detail why the use of high concentration LiFSI or LiTFSI‐based electrolytes can inhibit the corrosion of Al collector.^[^ ^54^ ^]^ Under the works of electrochemical force, Al^3+^ will be generated on the collector surface in these two ways: Al_2_O_3_ → 2Al^3+^ + 3/2O_2_ + 6e^−^ and Al → Al^3+^ + 3e^−^. As shown in Figure 9b–d,iIn LiPF_6_‐based electrolyte, F^−^ from ${{\text{PF}}_{6}}^{-}$ thermal decomposition or electrochemical decomposition can strongly bond with Al^3+^ or Li^+^, and the generated AlF_3_ and LiF are barely soluble in organic solvents, so the corrosion of Al is significantly inhibited. In dilute LiFSI‐based electrolyte, due to the existence of considerable free solvent molecules, Al^3+^ produced on the surface of Al quickly solvates with solvent molecules or complexes with anions to form solid substances (Al(FSI)_3_ or [Al(FSI)_x_]^3−x^), but the strong solvation ability of the solvent will dissociate these complexes. Finally, Al^3+^ will diffuse from the surface to the bulk electrolyte, which promotes the aforementioned two reactions to move to the right, that is, accelerating the dissolution of Al. However, in the high concentration LiFSI‐based electrolyte, almost all the solvents coordinate with Li^+^ and there is no free solvent molecule. After forming the same product as the thin electrolyte, the high concentration electrolyte cannot readily solvate Al^3+^ and dissociate the solid substance, so the aforementioned reaction is inhibited and the corrosion of Al is alleviated.

**Figure 10:**
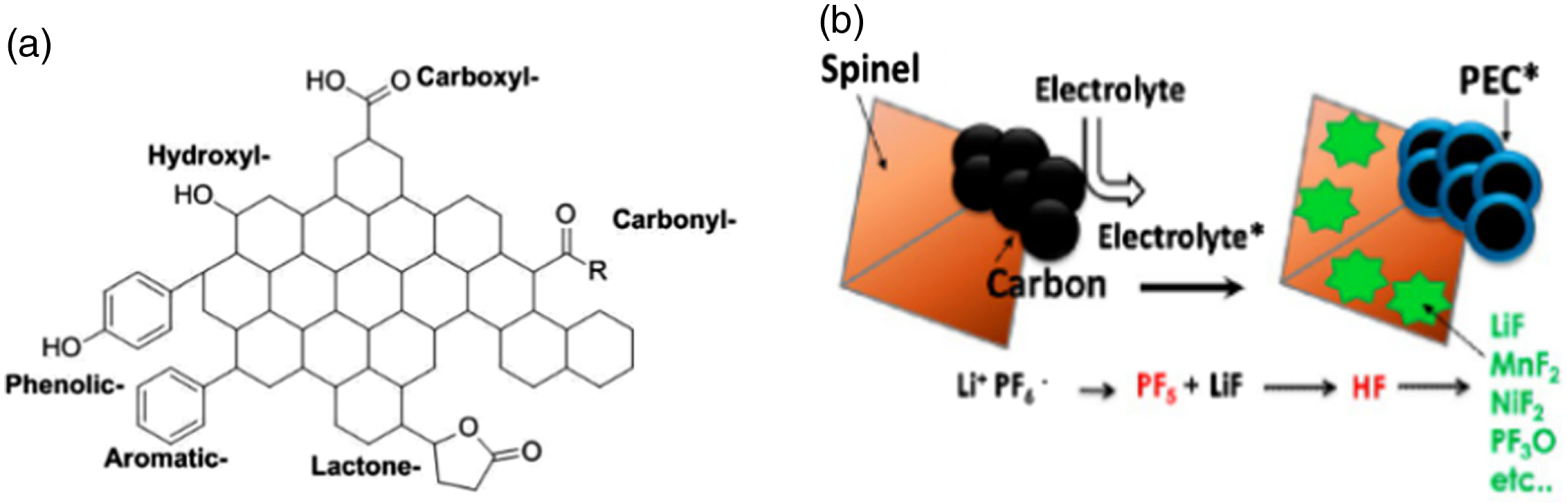
a) Schematic diagram of possible functional clusters around conductive carbon. b) Schematic diagram of parasitic reactions of electrolyte and conductive carbon at high voltage. a) Reproduced with permission.^[^ ^55^ ^]^ Copyright 2014, Institute of Physics Publishing. b) Reproduced with permission.^[^ ^56^ ^]^ Copyright 2013, American Chemical Society.

Conductive carbon is widely used to connect active materials and is an essential component for the synthesis of cathode pieces. Although the proportion of conductive carbon in the mass of the cathode is very low, only about 10%, its surface area is much higher than the active material in the cathode. Although conductive carbon itself does not contain any active chemical components, some oxygen‐containing functional groups, such as carbonyl, carboxyl, lactone, and hydroxyl, are inevitably introduced during manufacturing and storage (**Figure** **10**a).^[^ ^55^ ^]^ These functional groups readily catalyze electrolyte decomposition at high voltages. In addition, at a voltage higher than 4.5 V, the insertion reaction of anions is another reaction that occurs on the conductive carbon. The formation of interfaces and gas products on the conductive carbon was also observed, which may be due to self‐discharge or deintercalation of anions, as shown in Figure 10b.^[^ ^56^ ^]^

Moreover, some work has also studied the stability of the separators and the binder under high‐voltage conditions. Conventional separators may be blocked or mechanically broken under high voltage.^[^ ^57^ ^]^ Therefore, some researchers have proposed strategies for separators modification.^[^ ^58^, ^59^ ^]^ The most commonly used binder in the production of positive pole pieces is PVdF, which is considered to be stable at high voltages. Now some researchers have proposed better binders to improve the high‐voltage performance of batteries.^[^ ^60^, ^61^ ^]^

## 3 Different Electrolyte Modification Strategies Improve High‐voltage Performance

Commercial lithium battery electrolytes are composed of solvents, lithium salts, and additives, and their performance is not satisfactory when used in high cutoff voltage lithium batteries. Electrolyte modification strategy can achieve satisfactory high‐voltage performance by reasonably adjusting the types and proportions of these three components.

### 3.1 High‐Voltage Electrolyte Solvent

For the moment, the solvents used in commercial electrolytes are mainly carbonate solvents, such as EC, DEC, DMC, and EMC. They are often composed of EC with a high dielectric constant (used to dissolve lithium salts) and the latter three low‐viscosity chain carbonic acids ester solvent (used to improve lithium‐ion mobility) composition. However, as the operating voltage of the battery increases (to about 4.5 V), the carbonates decompose and the solid electrolyte film at the electrode interface becomes thicker and more uneven, so they are considered unsuitable for operating at excessive voltages. Therefore, people try to use other high‐voltage‐resistant solvents to replace carbonate solvents. This can be a complete substitution or partial substitution, because not only the priority of different solvent molecules participating in the reaction should be considered, but also the physical properties of the mixed solvent should be considered, such as conductivity, viscosity, and dielectric constant. The most common alternative solvents can be divided into fluorinated solvents, sulfone solvents, ionic liquids, and so on. Due to the strong electronegativity of fluorine atoms and strong electron‐withdrawing ability, the HOMO and LUMO energy levels of fluorinated solvents are lower than traditional carbonate solvents, that is, the anti‐oxidation and anti‐reduction ability of fluorinated solvents both are better than carbonate‐based solvents. Therefore, fluorinated solvents, such as fluoroethylene carbonate (FEC), difluoroethylene carbonate, trifluoropropylene carbonate, trifluoroethyl methyl carbonate, and ethyl 2,2,2‐trifluoroethyl carbonate are often used as high–voltage‐resistant solvents and SEI film‐forming additives. He et al. compared the oxidation potentials of different fluorinated solvents with linear sweep voltammetry, and the results showed that most of the systems replaced by fluorinated solvents had better antioxidant capacity than the common EC/EMC systems.^[^ ^62^ ^]^

**Figure 11:**
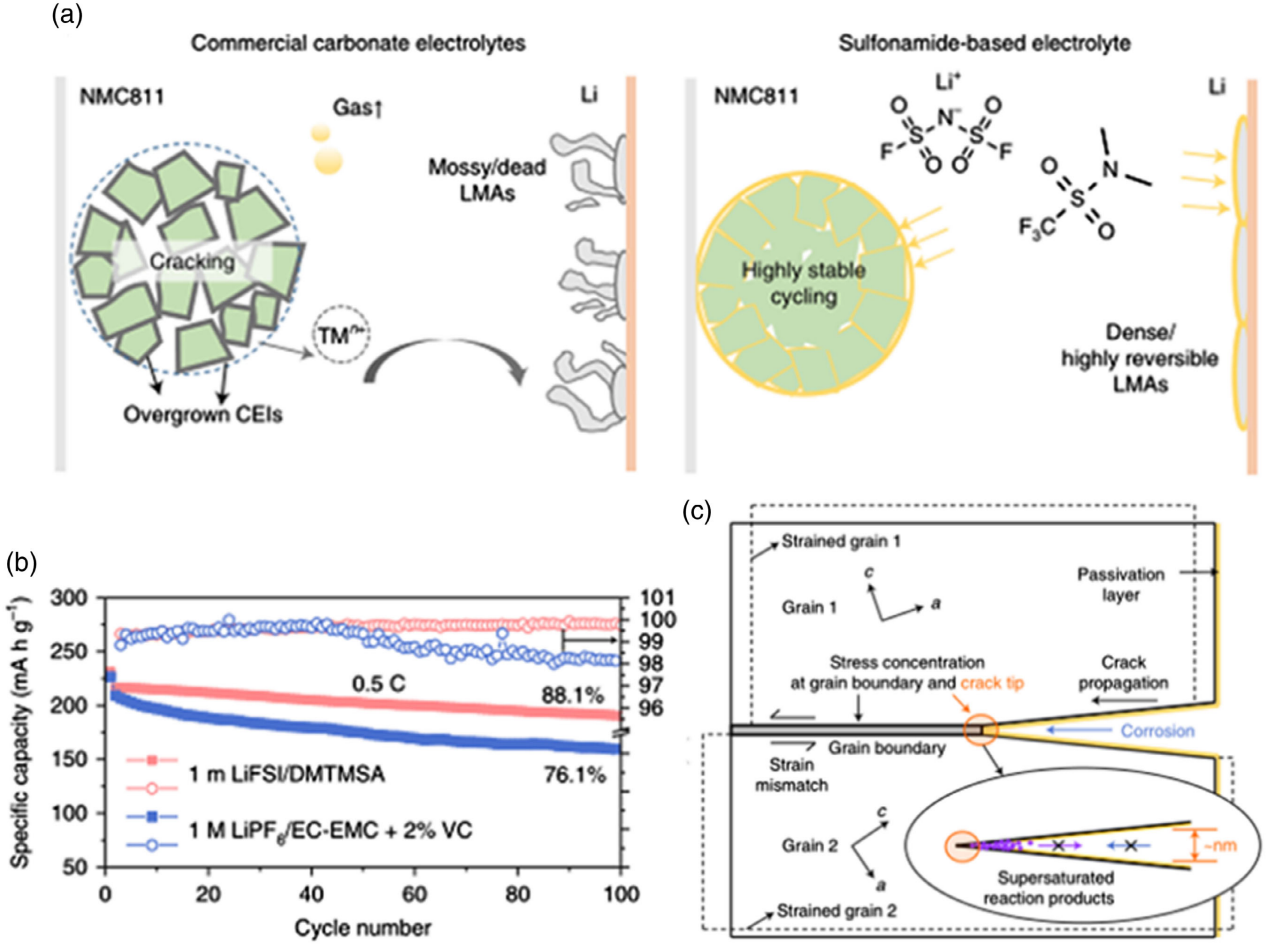
a) Schematic diagram of the difference between sulfonyl solvents and conventional commercial electrolytes; b) Specific capacity and CE of batteries with different electrolytes; c) Schematic diagram of the stress corrosion cracking (SCC) mechanism of a polycrystalline cathode and its suppression by limiting the solubility of reaction products in the liquid electrolyte. Reproduced with permission.^[^ ^65^ ^]^ Copyright 2021, Springer Nature.

The HOMO level of sulfoxide solvents is also lower than that of conventional carbonate solvents. Moreover, it has higher electrochemical stability, which is attributed to the more electronegativity of sulfonyl group than carbonyl group, resulting in an electrochemical window of more than 5 V. Sulfone solvents have strong interactions with lithium salts to achieve high conductivity, and have lower combustibility than carbonate solvents, so they are a promising high‐voltage solvent. Su et al. used the synthesized novel fluorinated sulfone as the high‐voltage NCM523 || graphite electrolyte, to improve its performance, compared with no fluoride sulfones electrolyte. The new synthesis of fluorinated sulfone showed stronger oxidation stability, lower viscosity, and better diaphragm invasive, making it a promising next‐generation high‐energy lithium‐ion battery electrolyte.^[^ ^63^ ^]^ Using sulfone as solvent alone usually can not meet the operating conditions, which is due to the high viscosity of sulfone solvent and may not be compatible with cathode and anode at the same time. Xue et al. found that the cycling performance of Li || LiNi_0.5_Mn_1.5_O_4_ half battery can be greatly improved by mixing sulfone and carbonate solvents as electrolyte compared with only sulfone as electrolyte. The capacity retention rate of the improved battery is still 97% after 100 cycles, while the capacity of the battery using only sulfoxide as electrolyte starts to rapidly decrease after 10 cycles.^[^ ^64^ ^]^ Xue and his colleagues proposed a new sulfonyl solvent (DMTMSA), the modification results of cathode and anode interphases can be briefly described in **Figure** **11**a.^[^ ^65^ ^]^ Using it with LiFSI in NCM811||Li batteries can achieve a capacity retention rate of 88% after 100 cycles at a high cutoff voltage of 4.7 V, the average Coulombic efficiency is higher than 99.65% (Figure 11b). After a series of analyses, they concluded that this solvent can effectively passivate the surface of cathode and anode at the same time, and can protect the Al current collector. Importantly, they found that the cracking of the cathode grains is not only a mechanical event, but also involves a chemical interaction between the surface and the electrolyte. This new view supports that their solvent can generate products in the cracks on the surface of the positive electrode material, preventing further penetration of the electrolyte and preventing parasitic reactions. The explanation of the mechanism is shown in Figure 11c.

Ionic liquid is a new type of organic solvent, which is mainly composed of organic cations and inorganic anions. Thanks to its physical and chemical properties that other solvents do not possess, such as low vapor pressure, nonflammability, high thermal stability, and electrochemical stability (oxidation potential is generally greater than 5 V), it is gradually being considered as a suitable high cutoff voltage Use and safe electrolyte. Yan et al. introduced a new electrolyte based on ionic liquid (PYR_14_‐TFSI) and fluorinated solvent (HFPM).^[^ ^66^ ^]^ HFPM can significantly reduce the viscosity of PYR_14_‐TFSI and increase the wettability of the separator. In addition, the coordination of PYR_14_‐TFSI and HFPM promotes the local dissolution of lithium nitrate. It is used in the assembly of Li || NCM622 batteries. After cycling 100 cycles at a rate of 0.5C at a cutoff voltage of 4.5V, the capacity retention rate is 94% and the Coulombic efficiency is 99.9%. It is worth noting that both ionic liquids and fluoroethers are nonflammable solvents. The flame‐retardant performance of this electrolyte is very excellent, which provides a new solution for the design of safe high‐energy lithium battery electrolytes. Although some ionic liquids have been used in high‐voltage lithium batteries, most ionic liquids have the properties of high viscosity and low conductivity, which makes the cycling performance worse, and the high melting point makes the ionic conductivity lower at low temperatures. Further research is needed to realize its practical application.

Organosilicon solvent is also considered to be a solvent suitable for high voltage, but it is rarely reported in lithium metal or lithium‐ion batteries, and is usually used in lithium–sulfur batteries.^[^ ^67^ ^]^ Its advantage is that it dissolves lithium salts well and is less toxic than carbonates. Zhang et al. synthesized a silane solvent called [3‐(2‐(2‐methoxyethoxy)ethoxy)‐propyl] triethoxysila (TESM2), which has an ionic conductivity of 1.14 mS cm^−1^ and an electrochemistry of up to 5.2 V window.^[^ ^68^ ^]^ With this solvent and 1 vol% vinyl carbonate (VC) as the electrolyte, the LiCoO_2_ || Li half‐cell can release a specific capacity of 153.9mAh g^−1^ (3.0–4.35 V, 28 mA g^−1^).

In addition to the solvents mentioned earlier, nitrile solvents with wide electrochemical windows and excellent thermal stability are also emerging in the field of high‐voltage solvents, which have previously been successfully used as plasticizers in polymer electrolytes.^[^ ^69^ ^]^ One of the most commonly used nitriles in electrolytes is succinonitrile (SN), which has a higher HOMO level than carbonate solvent, can induce the formation of a protective interface layer on the surface of high‐voltage cathode materials, and can remove hydrofluoric acid to slow transition metal dissolution on the cathode surface. However, as with most nitriles, SN has severe corrosion on the lithium metal cathode and needs to be mixed with a solvent that is easy to film on the anode. Zhang et al. used FEC mixed with SN (FEC: SN = 1:4 by weight) as the solvent for NCM523 || Li batteries. After 100 cycles at a high cutoff voltage of 4.7 V, the capacity retention rate was as high as 73.6%, while the commercial electrolyte was only 35.5%.^[^ ^70^ ^]^ This is mainly due to the formation of nitrogen‐containing, homogeneous, and highly ionic conductive CEI on the surface of the NCM cathode material after the use of this optimized electrolyte. Hu and his colleagues reported a dianion deep eutectic solution (D‐DES) based on a combination of SN and a functional lithium salt.^[^ ^71^ ^]^ SN, LiDFOB, and LiTFSI are all solid at room temperature. When they are mixed together, the mixture becomes liquid due to the strong interaction between molecules. The Li || Li‐battery using this D‐DES electrolyte can be cycled stably for 1 year (>10 000 h) at a current density of 2.5 mAh cm^−2^. After charging and discharging for 500 cycles of LiCoO_2_||Li battery at a high charging voltage of 4.7 V, the capacity retention rate still exceeded 70%. Surprisingly, its flame‐retardant performance is so superior that even after the pouch‐type battery is punctured or even cut, it can still be discharged without catching fire.

### 3.2 High‐Voltage Electrolyte Additive

Compared with other methods of improving high‐voltage batteries, the method of using high‐voltage additives has less dosage, low cost, simple method, and obvious effect, so it has been widely concerned. The HOMO level of most electrolyte additives is lower than that of solvent molecules, so they can preferentially participate in electrochemical reactions, forming a high‐quality protective film on the surface of the cathode to inhibit the decomposition of electrolytes and reduce the effect of parasitic reactions. At present, high‐voltage electrolyte additives can be briefly divided into several categories. All of them can effectively improve the high‐voltage cycle capacity of the battery. The difference is that the composition of CEI generated by their priority decomposition is different.

#### 3.2.1 Boron Additive

**Figure 12:**
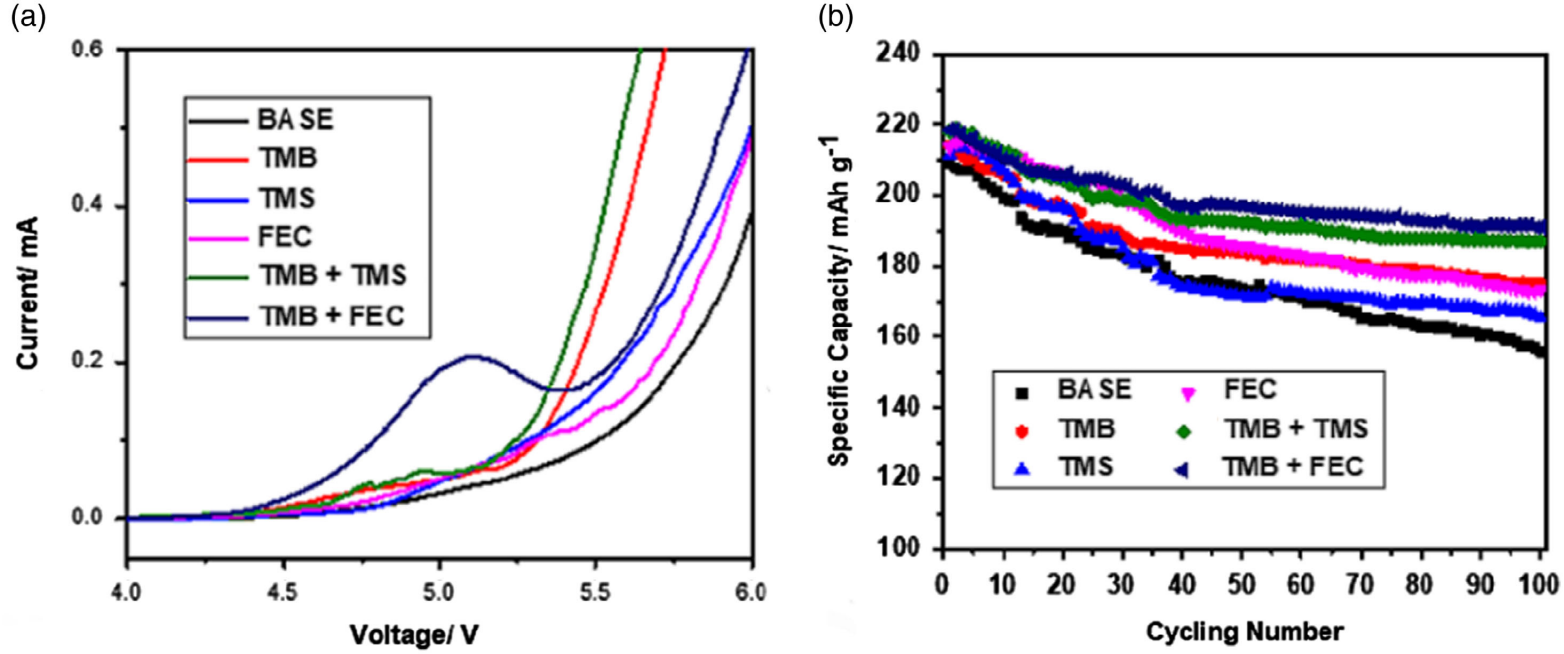
a) Linear sweep voltammetry (LSV) profiles of different electrolytes from the OCV to 6 V at 0.1 mV s^−1^: b) Cycling performance of the NCM811 || Li cells from 2.8 to 4.5 V at 0.1 C using different electrolytes. Reproduced with permission.^[^ ^74^ ^]^ Copyright 2021, American Chemical Society.

Boron additives are high‐voltage additives with excellent film‐forming effects. Some of these additives (such as LiBOB, LiDFOB) can also be used as lithium salts. Furthermore, a new borate‐based lithium salt was found to improve the stability of high‐voltage lithium batteries and protect Al current collectors from corrosion.^[^ ^72^ ^]^ Li et al. newly developed three lithium difluoro‐2‐fluoro‐2‐alkyl‐malonatoborate salts LiDFMFMB, LiDFEFMB, and LiDFPFMB as additives to improve the high‐voltage cycle performance of commercial EC/DMC/DEC (1:1:1 by volume) systems on batteries based on high‐voltage LNMO cathodes.^[^ ^73^ ^]^ Only 0.05 m additives are needed to form an effective passivation layer on the surface of the LNMO cathode, and the electrolyte containing the additives can significantly inhibit the co‐intercalation of the solvent into the graphite anode during the first cycle. The small impedance after the cycle and the small leakage current under the high voltage of 5 V can support the excellent properties of the film formed by the additive. SEM and XPS showed that a uniform and dense solid electrolyte interface was formed on the surface of graphite anode and LNMO cathode, respectively, after the addition of additives, which also indicated that the battery with an electrolyte containing additives had high Coulombic efficiency in the first cycle and good cycling stability. At the same time, after the CV cycling, the corrosion of Al in the electrolyte containing additives is more mild, while the corrosion of Al in the electrolyte without additives is much more serious. Liu et al. proposed two dual‐additive solutions, using the synergistic effects of trimethyl borate (TMB) with tetramethylene sulfone (TMS) and FEC, respectively, to protect the NCM811 cathode and LCO cathode at high potential.^[^ ^74^ ^]^ The difference between this work and other double‐additive methods is that, whereas other double‐additive methods basically have two additives acting independently, here there is an interaction between the two additives. When TMB and TMS exist in the electrolyte at the same time, the decomposition products of TMB can catalyze the oxidation of TMS and reduce the oxidation potential of TMS. When TMB and FEC are added at the same time, since the boron atom in TMB is electron‐deficient, it tends to coordinate with electron‐rich fluorine, which has been confirmed by Fourier transform infrared (FTIR) spectroscopy. It is speculated that the interaction between TMB and FEC can form a complex in the electrolyte, and the complex will be oxidized in advance to form CEI. (**Figure** **12**)

#### 3.2.2 Phosphorus Additive

**Figure 13:**
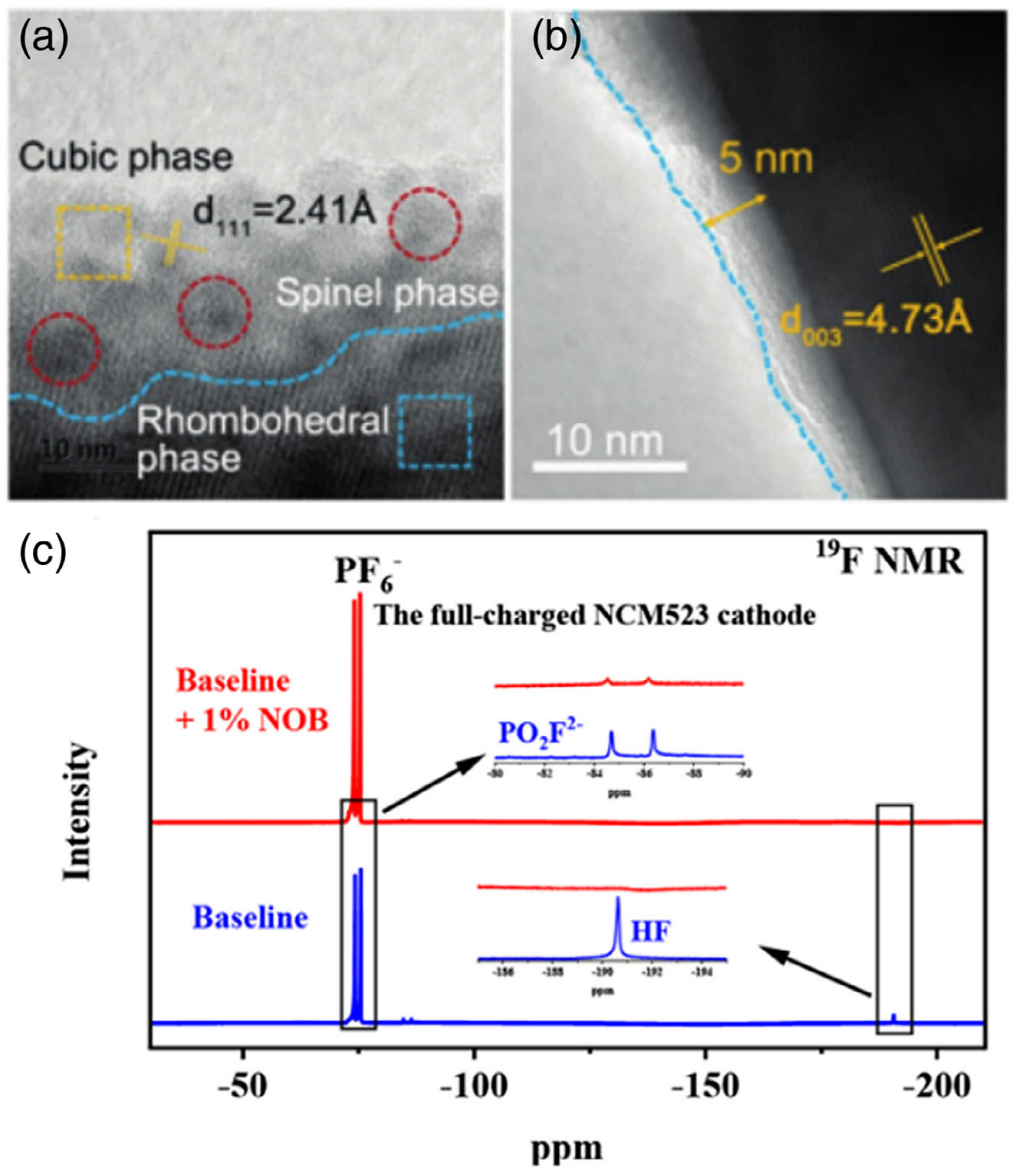
a) High‐resolution transmission electron microscopy images of NCM811 cycled in FEC/EMC electrolyte; b) ADV 1 wt% TPFPB and 3 wt% LiNO_3_ in 1M LiPF_6_‐FEC/EMC electrolyte; and c) ^19^F nuclear magnetic resonance (NMR) spectra of different electrolytes. The cathode was taken out from a fully charged battery and immersed in different electrolytes at 45 °C for 72 h. a,b) Reproduced with permission.^[^ ^76^ ^]^ Copyright 2020, Wiley‐VCH Verlag GmbH & Co. KGaA. c) Reproduced with permission.^[^ ^79^ ^]^ Copyright 2021, American Chemical Society.

Phosphorus additives are generally suitable as flame‐retardant functional additives, and are currently being developed as high‐voltage additives.^[^ ^75^ ^]^ Li et al. reported an advanced dual‐additive electrolyte that contains a unique solvation structure. The electrolyte is made of 1M LiPF_6_‐FEC/EMC (3:7 by volume) as the basic electrolyte, to which 3% LiNO_3_ and 1% tris (pentafluorophenyl)phosphine (TPFPP) or tris(pentafluorophenyl)borane (TPFPB) are added as an improved electrolyte.^[^ ^76^ ^]^ LiNO_3_ is an effective additive that can form a film on the anode, which can greatly improve the performance of lithium metal batteries, but its solubility in carbonate solvents is very limited. The added TPFPP (or TFPPB) is electron‐deficient, and at moderate heating temperatures, it seems to be the center of destroying LiNO_3_ clusters and manipulating solvation. Supported by the synergistic action of the two additives, the NCM811 || Li cell can circulate 140 cycles with a cutoff voltage of 4.5 V under a small amount of electrolyte, and the capacity retention rate is 80%, and also matched with LCO and LNMO cathode. After the cycle, an obvious layered‐spinel rock‐salt phase is formed on the surface of NCM811 cathode in the blank electrolyte, indicating that the reduction of unstable Ni^4+^ generates Ni^2+^, while the cathode in the dual‐additive effectively maintains the rhombohedral phase under the protection of ultrathin CEI of about 5nm, as shown in **Figure** **13**a,b. More surprisingly, only a very small amount of the electrolyte is required to maintain the long cycle of the battery, and when a small amount of blank electrolyte is used, the battery will fail early because the electrolyte is exhausted. Ji et al. synthesized pentafluorocyclotriphosphazene (PFPN) as a bifunctional additive in the electrolyte.^[^ ^77^ ^]^ On a LiCoO_2_ || Li battery with a cutoff voltage of 4.5 V, the capacity retention rate after 300 cycles is 91%, while the basic electrolyte is only 67%. In addition, in the presence of PFPN, the exotherm of the corresponding thermal reaction can be reduced, all due to the formation of uniform and dense CEI on the surface of LiCoO_2_ by the decomposition products of PFPN. Diethyl (thiophen‐2‐ylmethyl) phosphonate (DRYP) was reported to be capable to increase the capacity retention rate of the LNMO battery from 18% to 85% after 280 cycles at the content of 0.5%, and at 60 °C in the charge–discharge range of 3–4.9 V.^[^ ^78^ ^]^ This is due to the formation of a stable high‐voltage cathode interface, and the electrolyte containing this additive has good thermal stability and anti‐combustion characteristics of phosphoric acid components.

#### 3.2.3 Silane Additives

Silane additives are considered to be effective high‐voltage additives, and their effectiveness is generally attributed to their ability to eliminate HF's attack on the cathode material, thereby reducing the occurrence of additional reactions. Hu and his colleagues reported a new electrolyte additive N,O‐bis(trimehylsilyl)‐trifluoroacetamide (NOB), which can make NCM523 || graphite batteries run stably at high voltage.^[^ ^79^ ^]^ The electrolyte containing 1 wt% NOB can increase the capacity retention rate of the battery from 38% to 73% after 100 cycles at a rate of 1C and a cutoff voltage of 4.5 V. It is found that NOB can eliminate HF in the electrolyte (Figure 13c), after the cathode charged is immersed in electrolytes, the HF content in blank electrolyte is higher. And NOB can interact with F ions, and the generated free radicals are preferentially oxidized at the cathode, and the reaction products simultaneously construct a solid electrolyte interphase containing N at the cathode and anode interface to achieve suppression of the occurrence of parasitic reactions. Lyu et al. used three trimethylsilyl malonates as electrolyte additives for LiNi_0.8_Co_0.15_Al_0.05_O_2_ || Li high‐voltage batteries.^[^ ^80^ ^]^ Among them, the electrolyte containing 5 wt% BTMSMFM showed the best capacity retention rate under the cutoff voltage of 4.5 V. Scanning electron microscopy (SEM) showed that the additive formed a thinner CEI on the surface of the LiNi_0.8_Co_0.15_Al_0.05_O_2_ (NCA) cathode. X‐ray diffraction (XRD) further showed that under the action of additives, the crystal structure of NCA after cycling is less different from the initial crystal structure of NCA.

#### 3.2.4 Nitrile Additive

**Figure 14:**
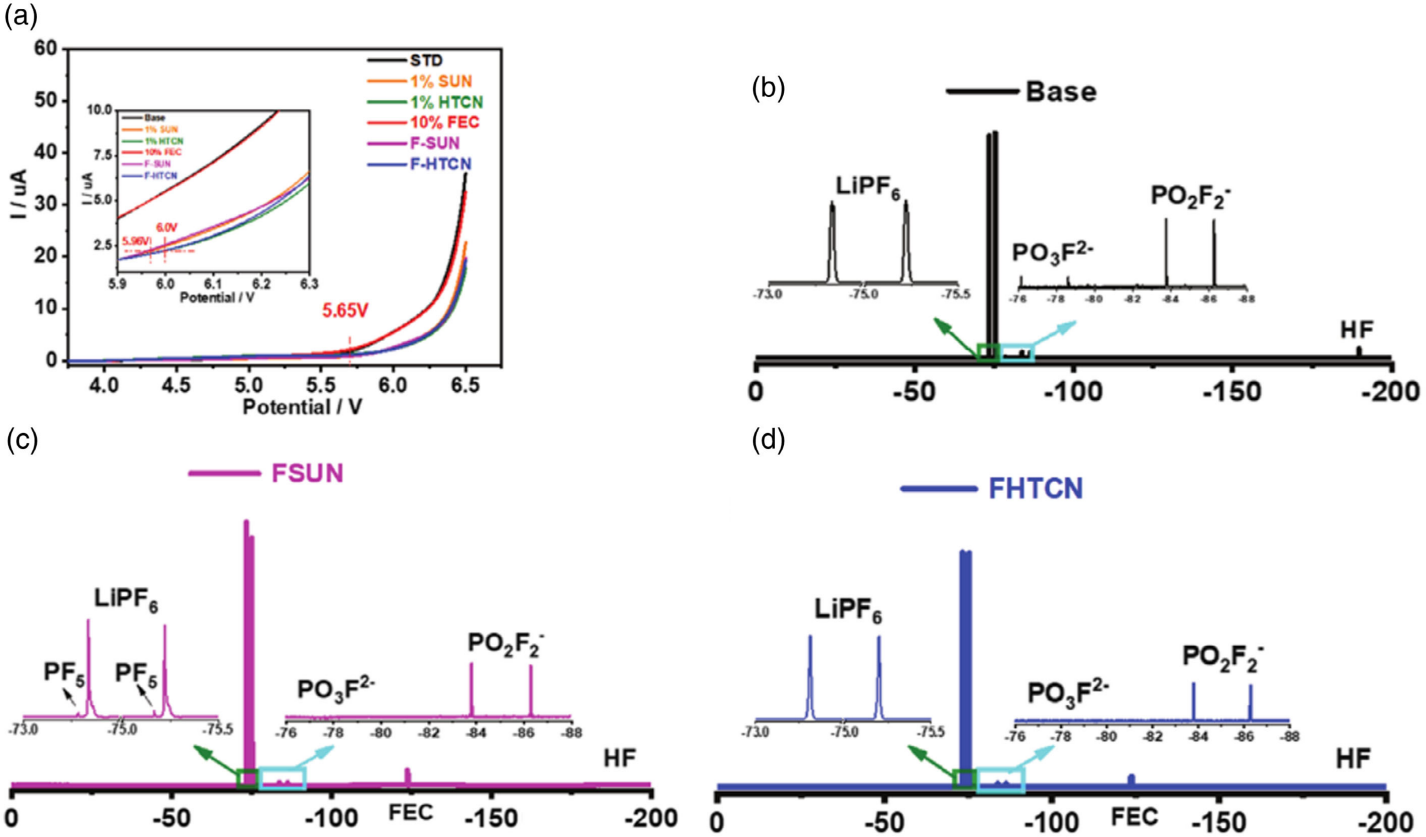
a) LSV curve of base electrolyte and electrolyte containing additives: b–d) ^19^F NMR spectra of the base electrolyte and the electrolyte containing additives. All electrolytes were stored at 55 °C for 7 days. Reproduced with permission.^[^ ^81^ ^]^ Copyright 2020, Wiley‐VCH GmbH.

In addition to being used as solvents, nitriles are often used as additives. Yang et al. reported a co‐additive of suberonitrile (SUN) or 1,3,6‐hexanetricarbonitrile (HTCN) and fluoroethylene carbonate (FEC).^[^ ^81^ ^]^ This additive can make the LiCoO_2_ || Li‐battery cycle for 300 cycles, and the capacity retention rate is still 72% at a high cutoff voltage of 4.6 V. In addition, the battery can be cycled for 500 cycles at a high current of 10C, while the capacity of the control group quickly drops to zero. After spectral analysis and theoretical calculations, they confirmed that the mechanism of action of nitriles is that the coordination of the lone pair of electrons on the N 2p orbital of the nitrile and the transition metal ion (Co^3+^/Co^4+^) is thermodynamically stable, reducing its oxidability and thereby reducing catalyze the decomposition of the electrolyte. The binding energy of SUN and HTCN with H_2_O is more negative than that of EMC with H_2_O and FEC with H_2_O, which shows that nitriles can effectively inhibit the hydrolysis of LiPF_6_. And HTCN has three ‐CN groups, which can effectively combine with two Li^+^, promote the dissociation of LiPF_6_ and inhibit the formation of PF_5_ (**Figure** **14**). Similar results were reported by Wang et al., who used fumaronitrile (FN) as an additive to enhance the performance of 4.5 V LiCoO_2_ || Li batteries.^[^ ^82^ ^]^ The protective cathode interface produced by FN protects the cathode material from the damage of HF, so that the structure of the LiCoO_2_ cathode can be maintained.

#### 3.2.5 Other Additives

In addition to the additives mentioned earlier, sulfone additives,^[^ ^83^, ^84^ ^]^ benzene derivative additives,^[^ ^85^ ^]^ and ionic liquid additives^[^ ^86^, ^87^ ^]^ have also been reported. The thickness of the film formed on the cathode material and the types of elements are variant, and the reaction mechanism is different. Therefore, it is worth looking forward to studying their internal mechanism of action and integrating many functions into one additive. As for how to select appropriate additives for a specific battery, theoretical calculations, experimental screening, and *pK*_*a*_ prediction are all commonly used methods.

### 3.3 High Concentration Electrolyte

If the concentration of lithium salt in the electrolyte is higher than 2 m, it can be called a high concentration electrolyte. In the conventional electrolyte, the solvent molecules can completely dissociate the lithium salt, and more than one solvent molecule is in the free state. Li^+^ is almost only coordinated with the solvent molecules, which is called the solvent separated ion pair (SSIPs) structure. The high concentration electrolyte has different solvation structures. With the increase of lithium salt concentration, all solvent molecules are used to dissociate lithium salt and participate in the coordination of Li^+^. At the same time, most anions also participate in the coordination of Li^+^, resulting in the formation of special solvation structures of contact ion pairs (CIPs) and cation–anion aggregates (AGGs). In this case, the anion is more carried by Li^+^ to the anode surface, and in the CIPs solvation structure, the whole LUMO of the electrolyte will transfer from the molecule of the solvent molecule to the anion and form an inorganic rich anion derivative film on the anode. Therefore, a high concentration electrolyte has been successfully used to inhibit the growth of lithium dendrite and improve the Coulomb efficiency of Li || Li half battery.^[^ ^88^ ^]^ Current research shows that high concentration electrolyte can also be applied to high‐voltage lithium battery system. As the salt concentration increases, the oxidation potential of the anion decreases, and more inorganic interfacial films are formed on the cathode interface.^[^ ^89^ ^]^ In addition, high‐concentration electrolytes are flame retardant better than conventional electrolytes, which meets safety requirements.^[^ ^90^ ^]^ By increasing the concentration of LiFSI to 10 m, Fan et al. not only achieved high Coulombic efficiency of stripping/intercalating lithium on the Cu electrode, but also successfully maintained the capacity of 86% of the original capacity on the 4.6 V NCM622 || Li‐battery after 100 cycles.^[^ ^91^ ^]^ After a series of characterization, they found that this result was due to the production of LiF‐rich SEI on the lithium anode, and the high‐concentration LiFSI protected the electrolyte from decomposing under high voltage and the Al current collector from being corroded. Ren et al. achieved a high‐voltage electrolyte in an ether solvent by increasing the concentration of LiFSI, which is generally a solvent unsuitable for use at high voltages because it decomposes at voltages above 4 V.^[^ ^92^ ^]^ As calculated by density functional theory, as the ratio of lithium salt molecules to solvent molecules increases, the HOMO energy level of the solvated complex of LiFSI‐DME decreases. This is due to the donation of lone electrons of oxygen atoms to the Li^+^ cation in the solvated complex has improved oxidation stability. The NCM111 || Li‐battery using the electrolyte can operate stably at a voltage of 4.5 V, although the improvement is not too great compared with commercial carbonate electrolyte, the realization of high‐voltage operation on ether solvents is a major breakthrough.

**Figure 15:**
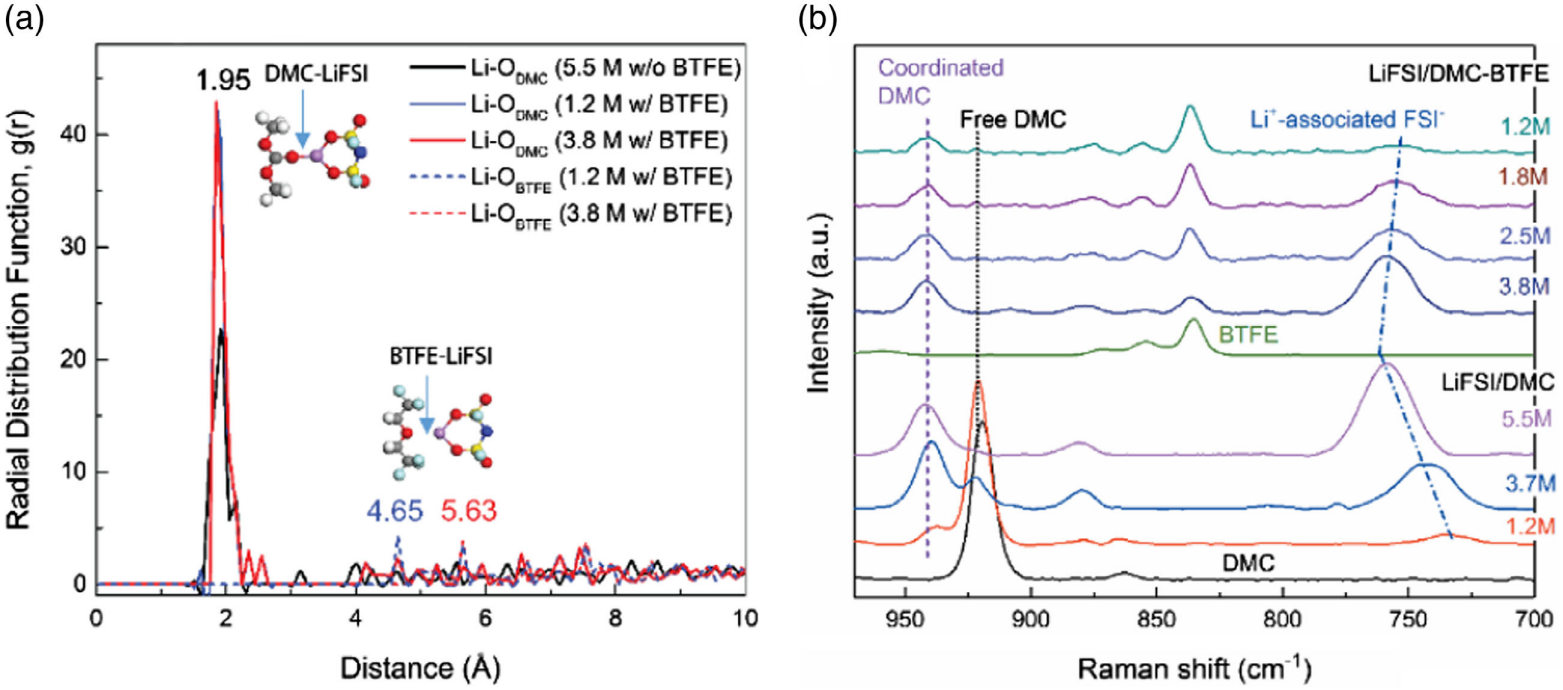
a) The radial distribution function of the Li‐O_DMC_ and Li‐O_BTFE_ pair calculated at 30 °C from ab initio molecular dynamics (AIMD) simulation trajectories. The inset shows the structure of DMC‐LiFSI and BTFE‐LiFSI; b) Raman spectra of different salt concentrations in pure DMC and various DMC/BTFE mixtures. Reproduced with permission.^[^ ^95^ ^]^ Copyright 2018, Wiley‐VCH.

Although the high‐concentration electrolyte has the advantages of high oxidation resistance, high carrier density, high Coulombic efficiency, inhibition of Al foil corrosion, and flame retardant. But it also has disadvantages, such as low conductivity and high cost. Therefore, a local high concentration strategy is proposed to make up for these deficiencies. In the local high concentration electrolyte, by introducing a diluent, the requirement on the lithium salt concentration can be reduced, and the cost can be reduced. The solvation structure in local high concentration electrolytes is similar to that in high concentration electrolytes, which is mainly dominated by CIPs. However, after adding diluent, reducing the salt concentration can achieve the same effect as or even better than that in high concentration electrolytes. Diluent molecules do not participate in the coordination of Li^+^ and are mainly distributed on the periphery of Li^+^ solvation shell. An ideal diluent should have very low solubility to lithium salt and high chemical stability. More importantly, the high viscosity and low conductivity of high concentration electrolytes can be improved by using low viscosity diluent, which greatly improves its practicability.^[^ ^93^ ^]^ In fact, as long as the solvation ability of the solvent is strong enough, it can be combined into local high concentration or high concentration, not only ether and carbonate solvents. In addition, there are no free solvent molecules in this system, the stability of the electrolyte is greatly improved, and the threshold for the formation of high concentration and local high concentration solvents is reduced. Matching with solvents with special functions can make up for the shortcomings of these solvents. For example, the mixed solution of triethyl phosphate (TEP) and bis (2,2,2‐trifluoroethyl) ether (BTFE) with local high concentration can achieve the functions of flame retardant and improve the cycling stability of the battery.^[^ ^94^ ^]^ After Chen et al. introduced BTFE as a diluent in DMC, only 1.2 m LiFSI was needed to achieve the same or even better results as higher concentrations of LiFSI.^[^ ^95^ ^]^ This electrolyte enables the NCM111 || Li‐battery to have a capacity retention rate of more than 80% after 700 cycles at a cutoff voltage of 4.3 V. They calculated that there are more DMC molecules around the re‐coordinated Li‐ion and fewer free DMC molecules (**Figure** **15**). Similar results were reported by Zhang et al.,^[^ ^96^ ^]^ but their cutoff voltages were all at or below 4.4 V. It is not high voltage in a way, so more research is needed on this development strategy.

## 4 Summary and Outlook

The desire to improve the battery life of electric cars and portable electronic devices is driving the development of high‐energy‐density lithium batteries. Increasing the cutoff voltage of lithium battery is an effective method to improve the specific capacity. However, with the increase of cutoff voltage, a series of problems come one after another, such as oxidation and decomposition of electrolyte, dissolution of transition metal ions, phase transition and surface rupture of cathode materials, etc. This eventually leads to a loss of reactive mass and lithium ions, as well as an increase in internal resistance and a decrease in capacity. To solve these problems, electrolyte modification methods have been widely studied. This review describes the causes of battery failure at high cutoff voltages, further describes how to use electrolyte modification strategies to improve the high‐voltage performance of batteries, and briefly introduces the related work in recent years.

All high‐voltage electrolyte modification methods can be roughly divided into three categories: high‐voltage solvents, high‐voltage additives, and high concentration or local high concentration electrolytes. At present, a lot of effort have been put into the development of high‐voltage electrolytes, but there are still many problems to be solved. Further research may be carried out from the following aspects in the future: 1) The electrochemical window of high‐voltage solvent is wider than that of traditional carbonate solvent, and the oxidation resistance is better. However, such solvents generally have the disadvantages of high viscosity and poor compatibility with electrodes and separators, and often need to be mixed with traditional carbonate solvents to cover their weaknesses. Therefore, it is significant to develop a high‐voltage solvent with low viscosity and good compatibility with electrodes and separators. 2) Adding additives to the electrolyte is a simple, low‐cost, and effective method to improve the performance of lithium batteries at high voltage, but the mechanism of action of most additives is still unclear. And an additive often has only one effect, cannot improve the performance of lithium batteries in many ways. The use of various additives at the same time will be incompatible and even lead to negative effects. Therefore, in future research, it is key to explore the mechanism of additives and develop multifunctional additives. 3) High concentration electrolyte has oxidation stability, low flammability, and low volatility, but its high viscosity, low ionic conductivity, high cost, so it is not ideal in the commercial aspect. The development of a method to reduce lithium concentration, such as local high concentration electrolyte, or the synthesis of a new cheap lithium salt is also of great research value. 4) To ensure stable operation of lithium battery under high voltage, it is necessary not only to withstand high‐voltage electrolyte and interface, but also to remove harmful products in the cycle process, such as reactive oxygen species. These new insights need to be discovered using more advanced characterizations, such as Cryo‐SEM, Spherical aberration electron microscopy, and Synchrotron Radiation. 5) Molecular dynamics calculations can help us simulate the reaction path in the electrolyte when the battery is charged and discharged, and also calculate the coordination of solvents and anions with Li^+^. Although it only plays an auxiliary role, it is very helpful for us to understand the mechanism of action and purposefully screening additives. 6) The high‐voltage performance of lithium batteries can be improved not only by electrolyte modification, but also by modification of cathode materials. Simultaneous electrolyte modification and cathode material modification, and using their synergistic effect to improve the high‐voltage performance of lithium batteries is a topic worth trying.

All in all, the development prospects of high‐voltage lithium batteries are very broad, and there are many problems they face, requiring great effort to invest in research. It is expected that this brief review can give some hints and inspiration to those engaged in the development of high‐voltage lithium batteries.

## Conflict of Interest

The authors declare no conflict of interest.

## References

[1] D. Larcher , J. M. Tarascon , Nat. Chem. 2015, 7, 19. 25515886 10.1038/nchem.2085

[2] B. Dunn , H. Kamath , J.‐M. Tarascon , Science 2011, 334, 928. 22096188 10.1126/science.1212741

[3] J. Huang , J. Liu , J. He , M. Wu , S. Qi , H. Wang , F. Li , J. Ma , Angew. Chem., Int. Ed. 2021, 60, 20717. 10.1002/anie.202107957 34288325

[4] F. Li , J. He , J. Liu , M. Wu , Y. Hou , H. Wang , S. Qi , Q. Liu , J. Hu , J. Ma , Angew. Chem., Int. Ed. 2021, 60, 6600. 10.1002/anie.202013993 33306226

[5] S. Qi , J. Liu , J. He , H. Wang , M. Wu , D. Wu , J. Huang , F. Li , X. Li , Y. Ren , J. Ma , J. Energy Chem. 2021, 63, 270.

[6] W. Xu , J. Wang , F. Ding , X. Chen , E. Nasybulin , Y. Zhang , J.‐G. Zhang , Energy Environ. Sci. 2014, 7, 513.

[7] D. Lin , Y. Liu , Y. Cui , Nat. Nanotechnol. 2017, 12, 194. 28265117 10.1038/nnano.2017.16

[8] H. Wang , J. He , J. Liu , S. Qi , M. Wu , J. Wen , Y. Chen , Y. Feng , J. Ma , Adv. Funct. Mater. 2021, 31, 2002578.

[9] S. Qi , J. He , J. Liu , H. Wang , M. Wu , F. Li , D. Wu , X. Li , J. Ma , Adv. Funct. Mater. 2021, 31, 2009013.

[10] S. Jiao , X. Ren , R. Cao , M. H. Engelhard , Y. Liu , D. Hu , D. Mei , J. Zheng , W. Zhao , Q. Li , N. Liu , B. D. Adams , C. Ma , J. Liu , J.‐G. Zhang , W. Xu , Nat. Energy 2018, 3, 739.

[11] J. He , H. Wang , Q. Zhou , S. Qi , M. Wu , F. Li , W. Hu , J. Ma , Small Methods 2021, 5, 2100441. 10.1002/smtd.202100441 34927858

[12] S. Qi , H. Wang , J. He , J. Liu , C. Cui , M. Wu , F. Li , Y. Feng , J. Ma , Sci. Bull. 2021, 66, 685. 10.1016/j.scib.2020.09.018 36654444

[13] K. Qin , K. Holguin , M. Mohammadiroudbari , J. Huang , E. Y. S. Kim , R. Hall , C. Luo , Adv. Funct. Mater. 2021, 31, 2009694.

[14] X. B. Cheng , R. Zhang , C. Z. Zhao , Q. Zhang , Chem. Rev. 2017, 117, 10403. 28753298 10.1021/acs.chemrev.7b00115

[15] M. Wu , Y. Li , X. Liu , S. Yang , J. Ma , S. Dou , SmartMat 2021, 2, 5.

[16] G. Jiang , F. Li , H. Wang , M. Wu , S. Qi , X. Liu , S. Yang , J. Ma , Small Struct. 2021, 2, 2000122.

[17] L. Cong , J. Liu , M. Armand , A. Mauger , C. M. Julien , H. Xie , L. Sun , J. Power Sources 2018, 380, 115.

[18] S.‐T. Myung , F. Maglia , K.‐J. Park , C. S. Yoon , P. Lamp , S.‐J. Kim , Y.‐K. Sun , ACS Energy Lett. 2016, 2, 196.

[19] J. W. Choi , D. Aurbach , Nat. Rev. Mater. 2016, 1, 16013.

[20] C. Yan , Y. X. Yao , X. Chen , X. B. Cheng , X. Q. Zhang , J. Q. Huang , Q. Zhang , Angew. Chem., Int. Ed. 2018, 57, 14055. 10.1002/anie.201807034 30094909

[21] C.‐C. Su , M. He , J. Shi , R. Amine , Z. Yu , L. Cheng , J. Guo , K. Amine , Energy Environ. Sci. 2021, 14, 3029.

[22] P. Wang , Y. Meng , Y. Wang , L. Chen , Z. Zhang , W. Pu , J. Li , C. Yang , D. Xiao , Energy Storage Mater. 2022, 44, 487.

[23] J. Y. Piao , L. Gu , Z. Wei , J. Ma , J. Wu , W. Yang , Y. Gong , Y. G. Sun , S. Y. Duan , X. S. Tao , D. S. Bin , A. M. Cao , L. J. Wan , J. Am. Chem. Soc. 2019, 141, 4900. 30827112 10.1021/jacs.8b13438

[24] Y. Wang , Q. Zhang , Z. C. Xue , L. Yang , J. Wang , F. Meng , Q. Li , H. Pan , J. N. Zhang , Z. Jiang , W. Yang , X. Yu , L. Gu , H. Li , Adv. Energy Mater. 2020, 10, 2001413.

[25] Y. Huang , Y. Zhu , H. Fu , M. Ou , C. Hu , S. Yu , Z. Hu , C. T. Chen , G. Jiang , H. Gu , H. Lin , W. Luo , Y. Huang , Angew. Chem., Int. Ed. 2021, 60, 4682. 10.1002/anie.202014226 33191621

[26] B. Chu , S. Liu , L. You , D. Liu , T. Huang , Y. Li , A. Yu , ACS Sustainable Chem. Eng. 2020, 8, 3082.

[27] J. B. Goodenough , Y. Kim , Chem. Mater. 2009, 22, 587.

[28] S. Tan , Y. J. Ji , Z. R. Zhang , Y. Yang , ChemPhysChem 2014, 15, 1956. 25044525 10.1002/cphc.201402175

[29] J. Li , L. E. Downie , L. Ma , W. Qiu , J. R. Dahn , J. Electrochem. Soc. 2015, 162, A1401.

[30] J. Wandt , A. T. S. Freiberg , A. Ogrodnik , H. A. Gasteiger , Mater. Today 2018, 21, 825.

[31] J. Self , C. P. Aiken , R. Petibon , J. R. Dahn , J. Electrochem. Soc. 2015, 162, A796.

[32] N. Laszczynski , S. Solchenbach , H. A. Gasteiger , B. L. Lucht , J. Electrochem. Soc. 2019, 166, A1853.

[33] T. Hatsukade , A. Schiele , P. Hartmann , T. Brezesinski , J. Janek , ACS Appl. Mater. Interfaces 2018, 10, 38892. 30335934 10.1021/acsami.8b13158

[34] F. Strauss , J. H. Teo , A. Schiele , T. Bartsch , T. Hatsukade , P. Hartmann , J. Janek , T. Brezesinski , ACS Appl. Mater. Interfaces 2020, 12, 20462. 32275815 10.1021/acsami.0c02872

[35] H. Zhao , J. Wang , H. Shao , K. Xu , Y. Deng , Energy Environ. Mater. 2022, 5, 327.

[36] M. Gauthier , T. J. Carney , A. Grimaud , L. Giordano , N. Pour , H. H. Chang , D. P. Fenning , S. F. Lux , O. Paschos , C. Bauer , F. Maglia , S. Lupart , P. Lamp , Y. Shao-Horn , J. Phys. Chem. Lett. 2015, 6, 4653. 26510477 10.1021/acs.jpclett.5b01727

[37] Y. Qian , S. Hu , X. Zou , Z. Deng , Y. Xu , Z. Cao , Y. Kang , Y. Deng , Q. Shi , K. Xu , Y. Deng , Energy Storage Materials 2019, 20, 208.

[38] B. L. D. Rinkel , D. S. Hall , I. Temprano , C. P. Grey , J. Am. Chem. Soc. 2020, 142, 15058. 32697590 10.1021/jacs.0c06363

[39] N.‐S. Choi , J.‐G. Han , S.‐Y. Ha , I. Park , C.‐K. Back , RSC Adv. 2015, 5, 2732.

[40] L. Xing , O. Borodin , G. D. Smith , W. Li , J. Phys. Chem. A 2011, 115, 13896. 22004044 10.1021/jp206153n

[41] M. Liu , J. Vatamanu , X. Chen , L. Xing , K. Xu , W. Li , ACS Energy Lett. 2021, 6, 2096.

[42] S. Klein , P. Harte , J. Henschel , P. Bärmann , K. Borzutzki , T. Beuse , S. van Wickeren , B. Heidrich , J. Kasnatscheew , S. Nowak , M. Winter , T. Placke , Adv. Energy Mater. 2021, 11, 2003756.

[43] Y. Y. Xia , M. Yoshio , J. Electrochem. Soc. 1996, 143, 825.

[44] T. Ohzuku , A. Ueda , J. Electrochem. Soc. 1994, 141, 2972.

[45] Z. Chen , Z. Lu , J. R. Dahn , J. Electrochem. Soc. 2002, 149, A1604.

[46] J. Vetter , P. Novák , M. R. Wagner , C. Veit , K. C. Möller , J. O. Besenhard , M. Winter , M. Wohlfahrt-Mehrens , C. Vogler , A. Hammouche , J. Power Sources 2005, 147, 269.

[47] J. Yang , Y. Xia , J. Electrochem. Soc. 2016, 163, A2665.

[48] P. Yan , J. Zheng , M. Gu , J. Xiao , J. G. Zhang , C. M. Wang , Nat. Commun. 2017, 8, 14101. 28091602 10.1038/ncomms14101 PMC5241805

[49] S. S. Zhang , J. Power Sources 2002, 109, 458.

[50] X. Zhang , T. M. Devine , J. Electrochem. Soc. 2006, 153, B375.

[51] S. S. Zhang , K. Xu , T. R. Jow , J. Electrochem. Soc. 2002, 149, A586.

[52] K. Matsumoto , K. Inoue , K. Nakahara , R. Yuge , T. Noguchi , K. Utsugi , J. Power Sources 2013, 231, 234.

[53] J. Wang , Y. Yamada , K. Sodeyama , C. H. Chiang , Y. Tateyama , A. Yamada , Nature Communications 2016, 7, 12032. 10.1038/ncomms12032 PMC4931331 27354162

[54] Y. Yamada , C. H. Chiang , K. Sodeyama , J. Wang , Y. Tateyama , A. Yamada , ChemElectroChem 2015, 2, 1687.

[55] X. Qi , B. Blizanac , A. DuPasquier , A. Lal , P. Niehoff , T. Placke , M. Oljaca , J. Li , M. Winter , J. Electrochem. Soc. 2014, 162, A339.

[56] N. P. W. Pieczonka , L. Yang , M. P. Balogh , B. R. Powell , K. Chemelewski , A. Manthiram , S. A. Krachkovskiy , G. R. Goward , M. Liu , J.‐H. Kim , J. Phys. Chem. C 2013, 117, 22603.

[57] R. Kostecki , L. Norin , X. Song , F. McLarnon , J. Electrochem. Soc. 2004, 151, A522.

[58] P. Zhai , K. Liu , Z. Wang , L. Shi , S. Yuan , J. Power Sources 2021, 499, 229973.

[59] J. Zhao , J. Zhang , P. Hu , J. Ma , X. Wang , L. Yue , G. Xu , B. Qin , Z. Liu , X. Zhou , G. Cui , Electrochim. Acta 2016, 188, 23.

[60] G. Li , Y. Liao , Z. He , H. Zhou , N. Xu , Y. Lu , G. Sun , W. Li , Electrochim. Acta 2019, 319, 527.

[61] H. Q. Pham , G. Kim , H. M. Jung , S.‐W. Song , Adv. Funct. Mater. 2018, 28, 1704690.

[62] M. He , C.‐C. Su , C. Peebles , Z. Zhang , J. Electrochem. Soc. 2021, 168, 010505.

[63] C.‐C. Su , M. He , P. C. Redfern , L. A. Curtiss , I. A. Shkrob , Z. Zhang , Energy Environ. Sci. 2017, 10, 900.

[64] L. Xue , K. Ueno , S.‐Y. Lee , C. A. Angell , J. Power Sources 2014, 262, 123.

[65] W. Xue , M. Huang , Y. Li , Y. G. Zhu , R. Gao , X. Xiao , W. Zhang , S. Li , G. Xu , Y. Yu , P. Li , J. Lopez , D. Yu , Y. Dong , W. Fan , Z. Shi , R. Xiong , C.‐J. Sun , I. Hwang , W.‐K. Lee , Y. Shao-Horn , J. A. Johnson , J. Li , Nat. Energy 2021, 6, 495.

[66] S. Yan , Y. Wang , T. Chen , Z. Gan , S. Chen , Y. Liu , S. Zhang , J. Power Sources 2021, 491, 229603.

[67] T. Liu , Z. Shi , H. Li , W. Xue , S. Liu , J. Yue , M. Mao , Y. S. Hu , H. Li , X. Huang , L. Chen , L. Suo , Adv. Mater. 2021, 33, 2102034. 10.1002/adma.202102034 34342060

[68] X. Qin , J. Wang , Y. Mai , D. Tang , X. Zhao , L. Zhang , Ionics 2013, 19, 1567.

[69] P. Hu , J. Chai , Y. Duan , Z. Liu , G. Cui , L. Chen , J. Mater. Chem. A 2016, 4, 10070.

[70] Q. Zhang , K. Liu , F. Ding , W. Li , X. Liu , J. Zhang , Electrochim. Acta 2019, 298, 818.

[71] Z. Hu , F. Xian , Z. Guo , C. Lu , X. Du , X. Cheng , S. Zhang , S. Dong , G. Cui , L. Chen , Chem. Mater. 2020, 32, 3405.

[72] B. Roy , P. Cherepanov , C. Nguyen , C. Forsyth , U. Pal , T. C. Mendes , P. Howlett , M. Forsyth , D. MacFarlane , M. Kar , Adv. Energy Mater. 2021, 11, 2101422.

[73] Y. Li , G. M. Veith , K. L. Browning , J. Chen , D. K. Hensley , M. P. Paranthaman , S. Dai , X.‐G. Sun , Nano Energy 2017, 40, 9.

[74] Q. Liu , G. Yang , S. Li , S. Zhang , R. Chen , Z. Wang , L. Chen , ACS Appl. Mater. Interfaces 2021, 13, 21459. 33905650 10.1021/acsami.1c04389

[75] Y. Li , Y. An , Y. Tian , H. Fei , S. Xiong , Y. Qian , J. Feng , J. Electrochem. Soc. 2019, 166, A2736.

[76] S. Li , W. Zhang , Q. Wu , L. Fan , X. Wang , X. Wang , Z. Shen , Y. He , Y. Lu , Angew. Chem., Int. Ed. 2020, 59, 14935. 10.1002/anie.202004853 32410377

[77] Y. Ji , P. Zhang , M. Lin , W. Zhao , Z. Zhang , Y. Zhao , Y. Yang , J. Power Sources 2017, 359, 391.

[78] Y. Zhu , X. Luo , H. Zhi , Y. Liao , L. Xing , M. Xu , X. Liu , K. Xu , W. Li , J. Mater. Chem. A 2018, 6, 10990.

[79] Z. Hu , K. Wang , Y. Che , M. Liu , W. Zhang , L. Xing , H. Wang , S. Li , X. Liu , W. Li , J. Phys. Chem. Lett. 2021, 12, 4327. 33929192 10.1021/acs.jpclett.1c00803

[80] H. Lyu , Y. Li , C. J. Jafta , C. A. Bridges , H. M. Meyer , A. Borisevich , M. P. Paranthaman , S. Dai , X.‐G. Sun , J. Power Sources 2019, 412, 527.

[81] X. Yang , M. Lin , G. Zheng , J. Wu , X. Wang , F. Ren , W. Zhang , Y. Liao , W. Zhao , Z. Zhang , N. Xu , W. Yang , Y. Yang , Adv. Funct. Mater. 2020, 30, 2004664.

[82] X. Wang , X. Zheng , Y. Liao , Q. Huang , L. Xing , M. Xu , W. Li , J. Power Sources 2017, 338, 108.

[83] J. Xiong , T. Zheng , Y. J. Cheng , J. Sun , R. Cao , Y. Xia , ACS Appl. Mater. Interfaces 2021, 13, 18648. 33860665 10.1021/acsami.1c00391

[84] X. Zheng , T. Huang , G. Fang , Y. Pan , Q. Li , M. Wu , ACS Appl. Mater. Interfaces 2019, 11, 36244. 31487984 10.1021/acsami.9b11795

[85] H. Lee , T. Han , K. Y. Cho , M. H. Ryou , Y. M. Lee , ACS Appl. Mater. Interfaces 2016, 8, 21366. 27509406 10.1021/acsami.6b06074

[86] W. Zhang , Q. Ma , X. Liu , S. Yang , F. Yu , RSC Adv. 2021, 11, 15091. 35424023 10.1039/d1ra01454d PMC8698397

[87] A. Tsurumaki , M. Branchi , A. Rigano , R. Poiana , S. Panero , M. A. Navarra , Electrochim. Acta 2019, 315, 17.

[88] Z. Peng , X. Cao , P. Gao , H. Jia , X. Ren , S. Roy , Z. Li , Y. Zhu , W. Xie , D. Liu , Q. Li , D. Wang , W. Xu , J. G. Zhang , Adv. Funct. Mater. 2020, 30, 2001285.

[89] X. Cui , J. Zhang , J. Wang , P. Wang , J. Sun , H. Dong , D. Zhao , C. Li , S. Wen , S. Li , ACS Appl. Mater. Interfaces 2021, 13, 59580. 34851095 10.1021/acsami.1c19969

[90] J.‐D. Xie , W.‐J. Liu , C. Li , J. Patra , Y. A. Gandomi , Q.‐F. Dong , J.‐K. Chang , Electrochim. Acta 2019, 319, 625.

[91] X. Fan , L. Chen , X. Ji , T. Deng , S. Hou , J. Chen , J. Zheng , F. Wang , J. Jiang , K. Xu , C. Wang , Chem 2018, 4, 174.

[92] X. Ren , L. Zou , S. Jiao , D. Mei , M. H. Engelhard , Q. Li , H. Lee , C. Niu , B. D. Adams , C. Wang , J. Liu , J.‐G. Zhang , W. Xu , ACS Energy Lett. 2019, 4, 896.

[93] X. Cao , H. Jia , W. Xu , J.‐G. Zhang , J. Electrochem. Soc. 2021, 168.

[94] S. Chen , J. Zheng , L. Yu , X. Ren , M. H. Engelhard , C. Niu , H. Lee , W. Xu , J. Xiao , J. Liu , J.‐G. Zhang , Joule 2018, 2, 1548.

[95] S. Chen , J. Zheng , D. Mei , K. S. Han , M. H. Engelhard , W. Zhao , W. Xu , J. Liu , J. G. Zhang , Adv. Mater. 2018, 30, 1706102. 10.1002/adma.201706102 29575163

[96] X. Zhang , L. Zou , Y. Xu , X. Cao , M. H. Engelhard , B. E. Matthews , L. Zhong , H. Wu , H. Jia , X. Ren , P. Gao , Z. Chen , Y. Qin , C. Kompella , B. W. Arey , J. Li , D. Wang , C. Wang , J. G. Zhang , W. Xu , Adv. Energy Mater. 2020, 10, 2000368.

[97] W. Li , B. Song , A. Manthiram , Chem. Soc. Rev. 2017, 46, 3006. 28440379 10.1039/c6cs00875e

[98] W. Li , A. Dolocan , P. Oh , H. Celio , S. Park , J. Cho , A. Manthiram , Nat. Commun. 2017, 8, 14589. 28443608 10.1038/ncomms14589 PMC5414066

[99] Z. Chen , J. R. Dahn , Electrochim. Acta 2004, 49, 1079.

[100] X. Lu , Y. Sun , Z. Jian , X. He , L. Gu , Y. S. Hu , H. Li , Z. Wang , W. Chen , X. Duan , L. Chen , J. Maier , S. Tsukimoto , Y. Ikuhara , Nano Lett. 2012, 12, 6192. 23170946 10.1021/nl303036e

[101] Q. Yang , W. Wang , K. Qian , B. Li , Adv. Mater. Interfaces 2019, 6, 1801764.

